# Oxygen transport kinetics underpin rapid and robust diaphragm recovery following chronic spinal cord injury

**DOI:** 10.1113/JP280684

**Published:** 2020-11-22

**Authors:** Philippa M. Warren, Roger W. P. Kissane, Stuart Egginton, Jessica C. F. Kwok, Graham N. Askew

**Affiliations:** ^1^ The Wolfson Centre for Age‐Related Diseases, Guy's Campus King's College London London UK; ^2^ School of Biomedical Sciences Faculty of Biological Sciences University of Leeds Leeds UK; ^3^ Institute of Life Course and Medical Sciences University of Liverpool Liverpool UK; ^4^ Institute of Experimental Medicine Czech Academy of Sciences Prague Czech Republic

**Keywords:** angiogenesis, capillary domain area, diaphragm, mechanical properties, spinal cord injury

## Abstract

**Key points:**

- Spinal treatment can restore diaphragm function in all animals 1 month following C2 hemisection induced paralysis. Greater recovery occurs the longer after injury the treatment is applied.
- Through advanced assessment of muscle mechanics, innovative histology and oxygen tension modelling, we have comprehensively characterized *in vivo* diaphragm function and phenotype.
- Muscle work loops reveal a significant deficit in diaphragm functional properties following chronic injury and paralysis, which are normalized following restored muscle activity caused by plasticity‐induced spinal reconnection.
- Injury causes global and local alterations in diaphragm muscle vascular supply, limiting oxygen diffusion and disturbing function. Restoration of muscle activity reverses these alterations, restoring oxygen supply to the tissue and enabling recovery of muscle functional properties.
- There remain metabolic deficits following restoration of diaphragm activity, probably explaining only partial functional recovery. We hypothesize that these deficits need to be resolved to restore complete respiratory motor function.

**Abstract:**

Months after spinal cord injury (SCI), respiratory deficits remain the primary cause of morbidity and mortality for patients. It is possible to induce partial respiratory motor functional recovery in chronic SCI following 2 weeks of spinal neuroplasticity. However, the peripheral mechanisms underpinning this recovery are largely unknown, limiting development of new clinical treatments with potential for complete functional restoration. Utilizing a rat hemisection model, diaphragm function and paralysis was assessed and recovered at chronic time points following trauma through chondroitinase ABC induced neuroplasticity. We simulated the diaphragm's *in vivo* cyclical length change and activity patterns using the work loop technique at the same time as assessing global and local measures of the muscles histology to quantify changes in muscle phenotype, microvascular composition, and oxidative capacity following injury and recovery. These data were fed into a physiologically informed model of tissue oxygen transport. We demonstrate that hemidiaphragm paralysis causes muscle fibre hypertrophy, maintaining global oxygen supply, although it alters isolated muscle kinetics, limiting respiratory function. Treatment induced recovery of respiratory activity normalized these effects, increasing oxygen supply, restoring optimal diaphragm functional properties. However, metabolic demands of the diaphragm were significantly reduced following both injury and recovery, potentially limiting restoration of normal muscle performance. The mechanism of rapid respiratory muscle recovery following spinal trauma occurs through oxygen transport, metabolic demand and functional dynamics of striated muscle. Overall, these data support a systems‐wide approach to the treatment of SCI, and identify new targets to mediate complete respiratory recovery.

## Key points

- Spinal treatment can restore diaphragm function in all animals 1 month following C2 hemisection induced paralysis. Greater recovery occurs the longer after injury the treatment is applied.
- Through advanced assessment of muscle mechanics, innovative histology and oxygen tension modelling, we have comprehensively characterized *in vivo* diaphragm function and phenotype.
- Muscle work loops reveal a significant deficit in diaphragm functional properties following chronic injury and paralysis, which are normalized following restored muscle activity caused by plasticity‐induced spinal reconnection.
- Injury causes global and local alterations in diaphragm muscle vascular supply, limiting oxygen diffusion and disturbing function. Restoration of muscle activity reverses these alterations, restoring oxygen supply to the tissue and enabling recovery of muscle functional properties.
- There remain metabolic deficits following restoration of diaphragm activity, probably explaining only partial functional recovery. We hypothesize that these deficits need to be resolved to restore complete respiratory motor function.

## Introduction

The ability to recover complete motor function following spinal cord injury (SCI) has long proved elusive, especially at protracted time points after the initial trauma (Shumsky *et al*. 2003). Although some experimental treatments have produced varying degrees of success, the mechanism to achieve rapid, total motor system recovery at these chronic stages is not yet fully realized. This is partly a result of current treatments overlooking the effects that such trauma has on peripheral muscle (in particular, its functional dynamics and morphology). SCI‐induced muscle paralysis causes atrophy and denervation, which can severely affect the degree to which any functional recovery is possible (Gill *et al*. 2014; Warren *et al*. 2018*b*). Even if neurological restoration occurs, without peripheral muscles operating optimally, functional restoration following injury will be minimal. It is imperative to understand how central and peripheral restoration can occur simultaneously to promote complete recovery of motor function within the clinic. Furthermore, understanding temporal muscular decline in response to chronic inactivity/paralysis, and the subsequent successful restoration of function, will provide a fundamental appreciation of the driving mechanisms important for muscle activity in a plethora of disease states.

Severe cervical SCI can cause a permanent paralysis of the diaphragm (Warren & Alilian, 2018; Warren *et al*. 2018*b*), increasing patient morbidity and mortality (Del Negro *et al*. 2018). Recovering motor function following these injuries is challenging, particularly at chronic stages (4+ weeks following trauma). Here, a combination of factors, including the formation of fibrotic and glial scaring, axonal dieback, and reduced regeneration‐associated gene and protein expression can mitigate recovery (Tran *et al*. 2018). Indeed, experimental treatments that are successful acutely after spinal injury seldom show similar effects chronically (Shumsky *et al*. 2003; López‐Vales *et al*. 2007) and often not all experimental animals respond to a specific treatment strategy (Lang *et al*. 2015; Warren *et al*. 2018*b*). We have provided evidence that restoration of diaphragm function following chronic SCI is possible through plasticity‐inducing mechanisms. Indeed, a single injection of chondroitinase ABC (ChABC) removed the inhibitory chondroitin sulphates (CSPGs) and enhanced plasticity of crossed projections within the respiratory phrenic motor pool (PMP) of the spinal cord sufficiently so that some diaphragm motor function was restored (Warren & Alilian, 2018; Warren *et al*. 2018*b*). Interestingly, this return of function occurred rapidly, despite prolonged paralysis causing significant diaphragm denervation (Warren *et al*. 2018*b*). However, the mechanism through which a paralysed and denervated hemidiaphragm can rapidly regain significant functionality to affect synchronized and stable breathing is unknown. Without understanding this mechanism, and determining whether functional muscle properties are completely restored following treatment, recovery in human patients will be limited because complete motor restoration may not be achieved.

Following injury or disease, modifications in the optimal conditions for muscle function and performance (Kissane *et al*. 2018*a*), vascular composition (Hauton *et al*. 2015*a*,*b*; Fowler *et al*. 2019; Tickle *et al*. 2020), and metabolic profile (Hauton *et al*. 2015*a*,*b*; Fowler *et al*. 2019) have caused profound alterations in the capacity of that muscle to function correctly. We hypothesized that injury‐induced chronic paralysis would cause significant alterations to diaphragm functional characteristics and morphology. Furthermore, it was proposed that ChABC‐mediated restoration of function occurs through the return of these characteristics to pre‐injury conditions.

No current assessments have been performed on the characteristics of the diaphragm following spinal induced recovery of muscle activity after prolonged injury‐induced paralysis. As such, the degree to which functional capacity of the diaphragm could affect recovery of motor activity has not been assessed. We aim to provide these data. Similarly, previous studies have shown only modest alterations to the diaphragm functional properties and/or histology following SCI‐induced paralysis (Metzger *et al*. 1985; Miyata *et al*. 1995; Mantilla *et al*. 2013*b*). This may be because the techniques used did not employ physiologically relevant assessments. To fully assess the degree to which the diaphragm is affected by SCI, we have used a unique interdisciplinary approach promoting physiological relevance that will determine changes in functional output of the diaphragm, capacity of the isolated muscle to produce and sustain power, structural alterations in the muscle fibre morphology, modifications in diaphragm microvascular supply, and model oxygen transport within the tissue (Altringham & Young, 1991; Warren & Alilian, 2018; Kissane *et al*. 2018*a*,*b*; Warren *et al*. 2018*b*). This combination of techniques and innovative protocols demonstrate how interacting responses of muscle fibre size, microvascular supply and tissue metabolic status can affect functional muscle capacity and motor system output following spinal injury, as previously suggested in chronic heart failure (Bowen *et al*. 2017), diabetes (Dunford *et al*. 2017) and ageing (Barnouin *et al*. 2017).

Here, we demonstrate that plasticity‐induced restoration of diaphragm activity following chronic and severe spinal injury can occur in all experimental animals. We establish that chronic diaphragm paralysis causes significant deficits in muscle functional properties. This concurrently alters both the global and local morphology of diaphragm tissue, impeding oxygen diffusion to the muscle and reducing functional capacity and activity. We demonstrate that ChABC‐mediated recovery of diaphragm activity can restore muscle functional kinetics, as well as adjust morphological parameters to facilitate oxygen supply to the tissue, which enables restoration of muscle functional characteristics. However, we demonstrate that deficits in metabolic capacity of the diaphragm remain following restoration of activity. Collectively, our data examine the mechanism of recovery within diaphragm tissue following spinal trauma, and allow for the potential identification of novel treatment targets to resolve underlying deficits.

## Methods

### Ethical approval

Experiments were approved by the University of Leeds Animal Welfare and Ethics Committee (70/8085), were conducted in accordance with UK Animals (Scientific Procedures) Act 1986 (ASPA), and conform to the principles and regulations as described in Grundy (2015). They have therefore been performed in accordance with the ethical standards laid down in the 1964 *Declaration of Helsinki* and its later amendments. At the time of data processing and analysis of all experiments and assessments, investigators were blind as to the treatment group of each animal.

**Figure A1:**
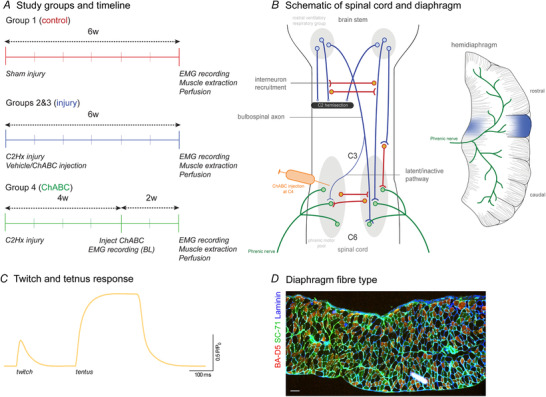
Injury model and experimental paradigm *A*, experimental protocol. *B*, schematic of inspiratory respiratory motor inputs to the diaphragm after a lateral C2 hemisection and image of a hemidiaphragm showing rostral section taken for muscle analysis (blue). *C*, representative trace of twitch and tetanus protocol. In a twitch, the stimulation is brief meaning that the muscle starts to relax before reaching peak force. If multiple contractions occur before complete relaxation of the muscle, the twitch summates. With a stimulus train the muscle reaches peak force and plateaus, causing tetanic contraction. *D*, representative figure showing heterogeneous fibre type distribution in the hemidiaphragm. Scale bar = 10 μm. [Color figure can be viewed at wileyonlinelibrary.com]

Animals were housed in groups of three or four under a normal 12:12 h dark/light photocycle at 21°C with free access to food, water and environmental enrichment *ad libitum*. The health and welfare of all animals was monitored daily by veterinary staff and the study investigators at the University of Leeds and was in accordance with the Animal Welfare Act 2006 and The Welfare of Farm Animals (England) Regulations 2007. Thirty‐two Sprague–Dawley female rats (weighing 263 ± 5.98 g; sourced from Charles River Laboratories, Margate, UK) were used in the present study. One animal stopped breathing immediately following the injury and could not be recovered with manual ventilation. Furthermore, one animal was removed from the study as a result of complications unrelated to the initial spinal injury or the experimental protocol. Six animals received ChABC treatment at either 1 or 12 weeks post‐injury and had EMG recordings alone (*n* = 3 each). The remaining 24 animals were divided into four groups (see Appendix, Fig. A1*A*): (i) sham injured animals (*n* = 12); (ii) C2 hemisected with immediate injection of saline vehicle (*n* = 4); (iii) C2 hemisected animals treated at the time of injury with ChABC (*n* = 3); and (iv) C2 hemisected animals treated four weeks following injury with ChABC (*n* = 5). Animals in groups 1–4 had EMG recordings, muscle kinematics and tissue collected for morphology 6 weeks following the initial injury/surgery (group 4 had an additional EMG at 4 weeks post injury prior to treatment application; see Appendix, Fig. A1*A*). Saline vehicle controls were not performed because it has been shown previously, at multiple time points, that the vehicle has no effect upon spinal plasticity, regeneration or respiratory motor recovery (Warren *et al*. 2018*b*). Animals were treated with ChABC for 2 weeks because we have shown previously that the degree of respiratory motor recovery engendered from this treatment does not significantly increase beyond this point. Indeed, animals assessed up to 6 months after treatment showed the same degree of recovery within the diaphragm as those 2 weeks after treatment (Warren *et al*. 2018*b*).

### Surgical procedures and treatment application

#### Spinal surgery

**Figure 1:**
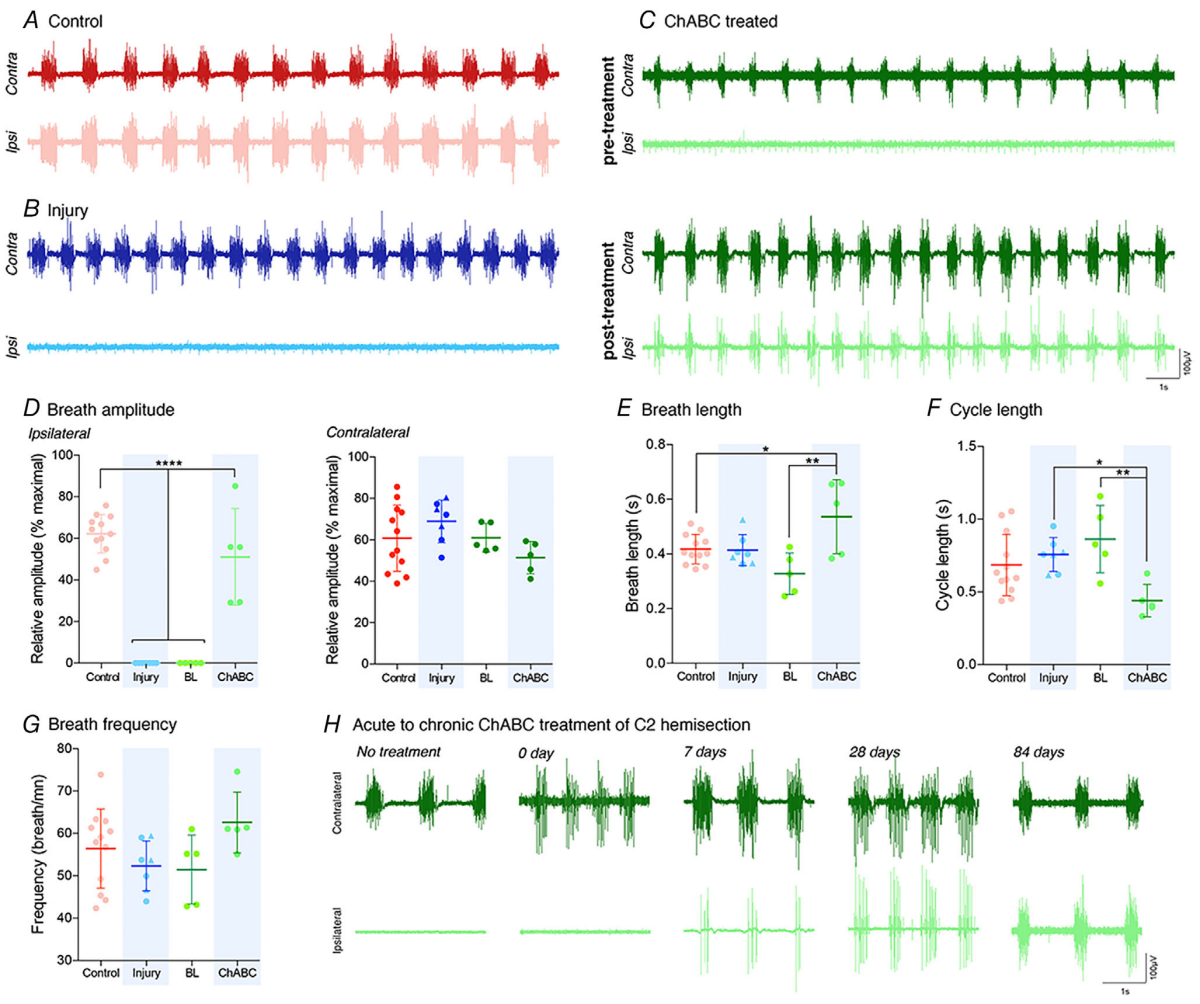
Induction of spinal plasticity restores ipsilateral hemidiaphragm function after cervical injury Data are shown from control (red; group 1), injury (blue; groups 2&3) and ChABC (green; group 4) animals. *A*–*C*, representative diaEMG recordings for control (red), injury (blue) and ChABC groups (green; both pre‐ (BL) or post‐treatment) (*n* = 5–12). All data presented are from the same animal. *D*, average amplitude of ipsilateral and contralateral diaEMG. *E–G*, ventilatory parameters showing (*E*) breath length, (*F*) cycle length and (*G*) breath frequency for whole diaphragm. For all graphs, triangle data points represent group 3 animals. For (*A*) to (*G*), pre‐treatment (BL) recordings of ChABC animals were conducted 2 weeks prior to the other recordings shown. *H*, representative diaEMG recordings of SCI animals treated with ChABC at varying time points from the time of injury (*n* = 3–9). Data obtained from groups 1, 3, 4 and independently treated animals. Ipsilateral and contralateral recordings are from the same animal at each time point. Data assessed via one‐ or two‐way ANOVA with the following sample sizes: control = 12, injury = 7, ChABC = 5. ^*^ *P* < 0.05, ^**^ *P* < 0.01 and ^****^ *P* < 0.0001. If no *post hoc* result is shown, the comparison was not significant. Values represent the mean ± SD. Abbreviations: Ipsi, ipsilateral; contra, contralateral hemidiaphragm recordings. [Color figure can be viewed at wileyonlinelibrary.com]

This spinal hemisection model (see Appendix, Fig. A1*B*), as well as its effects on respiratory function, has been fully characterized previously (Warren *et al*. 2018*b*). Animals were anaesthetized with Isoflurane (5%; Zoetis, UK Ltd, Leatherhead, UK) at 2 L min^−1^ O_2_ flow (0.4 L min^−1^ kg^−1^) induction, and maintained with 2.5% anaesthetic (0.20 L min^−1^ kg^−1^) throughout surgery. Depth of anaesthesia was assessed throughout surgery through continuous monitoring of the pedal reflex, respiration rate and pattern, and colour of mucous membranes. Upon reaching a surgical plane of anaesthesia, the dorsal neck was shaved and cleansed and the animals were given a s.c. injection of Vetergesic (Ceva Animal Health Ltd, Amersham, UK) (30 μg kg^−1^). Body temperature was maintained throughout the surgery at 37 ± 1°C using a homothermic heat pad (Harvard Apparatus, Cambridge, MA, USA). Following completion of a dorsal midline incision from C1–C3, the skin and paravertebral muscles were retracted and a laminectomy performed over C2 exposing the spinal cord. If requiring a spinal injury (groups 2 and 3) (Fig. 1 *A*) a C2 durotomy and hemisection was performed using a 21 G needle at the level of the dorsal roots and completeness confirmed through microscopy (Warren *et al*. 2018*b*). Muscle layers were sutured together (3‐0 Vicryl; Ethicon Inc., Somerville, NJ, USA) and the skin closed using wound clips. Animals were given s.c. injections of saline and recovered in a heated environment before transfer to their home cage. Analgesia and hydration were maintained for 5 days post‐surgery in addition to nutritional support if the animal's weight fell 5% below that determined pre‐injury. No animal showed any adverse effects to the surgery or procedures performed.

#### Injections

Animals receiving EMG recordings along with those in groups 3 and 4 received an injection of ChABC (Merck; 20 U ml^−1^) at the time points specified in the text. Animals were anaesthetized with isoflurane, the dorsal neck shaved and cleansed, and Vetergesic (30 μg kg^−1^; s.c.) administered. Body temperature was maintained at 37 ± 1°C using a homothermic heat pad (Harvard Apparatus). Following a dorsal midline incision from C3–C5, a laminectomy was performed over C4 and the most rostral section of C5 and the dura cut. A pulled glass pipette attached to a Pneumatic PicoPump (World Precision Instruments, Sarasota, FL, USA) was stereotaxically placed at the C4 phrenic nucleus (1.1 mm left of midline and 1.6 mm ventral from the spinal cord dorsal surface) at the level of the dorsal roots. After placement and a 5 min rest period, 350 nl of ChABC was injected into the spinal cord followed by a 5 min rest period. With the pipette removed, the incision was closed by suturing the muscle layer and through the use of wound clips on the skin. Analgesia and hydration were maintained for 5 days post‐surgery.

### Diaphragm EMG recordings

DiaEMG recordings occurred either prior to injection of ChABC or extraction of the diaphragm muscle at the experimental end‐point. In the former, animals were anaesthetized with isoflurane (2.5%; 0.20 L min^−1^ kg^−1^); in the latter condition, animals were anaesthetized with ketamine/xylazine cocktail (70 mg kg^−1^/7 mg kg^−1^) through i.p. injection. The animal's abdomen was shaved and cleansed. Body temperature was maintained at 37 ± 1°C using a homothermic heat pad (Harvard Apparatus). Following a 4 cm laparotomy, bipolar platinum electrodes (Grass Technology, Middleton, WI, USA) were placed in the costal left and right hemidiaphrams, dorsal to the anterolateral branch of the inferior phrenic artery (Warren & Alilian, 2018; Warren *et al*. 2018*a*,*b*). Recordings occurred during 10 min eupnoea, and a 20 s nasal occlusion (time determined due to ethical considerations). DiaEMG activity was amplified (gain 5000×), band pass filtered (30–3000 Hz; Grass Technology), digitized and recorded (CED1401; Spike2; Cambridge Electronic Design, Cambridge, UK). The integrated signal was rectified and smoothed with a time constant of 0.075 s. Following recording in recovery animals, Vetergesic and saline was administered s.c. and the incision site closed. Analgesia and hydration were maintained for 5 days post‐surgery.

Recordings were assessed in 30 s time windows to determine breathing frequency, amplitude, and cycle time. The occlusion performed during the diaEMG recording induces a reproducible motor recruitment and drive to the respiratory motor system (averaged over three breaths) which can be used to determine activity of the diaphragm relative to a constant that is not altered by slight changes in electrode placement, fibrosis or depth of anaesthesia (Mantilla *et al*. 2011; Felix *et al*. 2014; Warren *et al*. 2018*a*,*b*). Animals reported as having no EMG signal from the hemidiaphragm exhibited this deficit in activity throughout all regions of the muscle (costal, crural and sternal). No animal was excluded from the study based upon its response to treatment.

DiaEMG recordings were made under light anaesthesia, which, although typical in the field (Mantilla *et al*. 2007; Warren & Alilian, 2018; Warren *et al*. 2018*b*), may have the potential to minimize respiratory motor output compared to the un‐anaesthetized animal. However, recordings were conducted under light isoflurane anaesthesia to minimize this effect (Bezdudnaya *et al*. 2018) and showed no spontaneous recovery in diaphragm function. Because other laboratories have reported spontaneous activity in the muscle under ketamine–xylazine anaesthesia (Mantilla *et al*. 2013*a*), which we do not show, it is expected that the recordings we report accurately reflect *in vivo* activity and any potential recovery within our system. No animal in group 3 showed any recovery in ipsilateral diaphragm activity. Because this experiment concerns muscle functional characteristics, and these animals displayed the same functional activities as those in group 2 and no statistical divergence from these animals in any condition, the results of these two groups have been pooled together. However, to ensure accuracy in reporting, the animals in group 3 have been represented in all figures as a triangle.

### Muscle functional properties

#### Diaphragm extraction and preparation

Following completion of the diaEMG recordings, the animal was given a terminal dose of pentobarbital sodium 20% (Pentoject; Animalcare Ltd, York, UK) prior to exsanguination in accordance with ASPA Schedule 1 requirements. The whole diaphragm was carefully dissected from the thoracic cavity and immediately placed in Krebs–Henseleit solution (in mmol l^−1^: 117 NaCl, 4.7 KCl, 2.5 CaCl_2_, 1.2 MgSO_4_, 24.8 NaHCO_3_, 1.2 KH_2_PO_4_ and 11.1 glucose) at 4 ± 0.2°C and oxygenated (95% O_2_, 5% CO_2_). The diaphragm was pinned out in a Petri dish at approximately resting length. A medial section of the costal diaphragm ∼2.5 mm in width was dissected out of each hemidiaphragm, being careful not to cut through muscle fascicles. Diaphragm mass was unavoidably variable between each preparation (Tallis *et al*. 2017) (mass 35–60 mg) and thus was accounted for in all calculations. One muscle section was used immediately, whereas the other was pinned at approximately resting length and stored at 4 ± 0.2°C, in oxygenated Krebs–Henseleit solution for ∼3 h (order of assessment was randomized). The muscle under assessment was suspended vertically in a flow‐through Perspex chamber filled with circulating, oxygenated Krebs–Henseleit solution at 37 ± 0.2°C. Muscle operating temperature affect contractile performance; therefore, experiments were conducted at this temperature to ensure data were collected under physiologically relevant conditions. At one end, stainless‐steel clips were attached to two ribs anchoring the dissected muscle in the chamber, whereas the central tendon at other end was attached to an ergometer (series 300B‐LR; Aurora Scientific Inc., Bristol, UK) via a lightweight (100 mg) stainless‐steel rod fastened to a moveable stage that controlled the muscle's resting length. Following mounting, the muscle preparation was left for 30 min to thermoequilibrate in fresh, oxygenated Krebs–Henseleit solution and to recover from the dissection. Parallel platinum electrodes were placed inside the chamber on either side (but not in contact with) the muscle. The length of the hemidiaphragm sections were controlled using custom‐written software (CEC Testpoint, version 7, CEC Capital Equipment, Norton, MA, USA), which was converted to an analogue signal through a 16‐bit A/D converter (DAS1802AO; Keithley Instruments, Beaverton, OR, USA) that controlled the length of the muscle via the ergometer. To assess the condition of the muscle preparation during the experiment and to enable correction for any decline in performance, control isometric contractions were performed after every fourth set of contractions and relative force calculated by assuming a linear decline in *P*_0_. Data were excluded (and the experiment terminated) if the achieved *P*_0_ fell below 70% of the initial value attained. A 5 min rest period occurred after each tetanic and isotonic contraction to allow for muscle recovery.

#### Diaphragm isometric contractile properties

Each muscle section was subjected to a series of isometric twitches (supramaximal stimulus with a 0.2 ms pulse width (Syme & Stevens, 1989; Altringham & Young, 1991) (see Appendix, Fig. A1*C*) and maximal isometric twitch force determined by varying the muscle length (0.5 mm incremental changes). The muscle length that generated the maximal twitch force was defined as the optimal length (*L*_0_) and was used in all subsequent experiments. Our calculated *L*_0_ was calculated in line with standard protocols and our control groups have similar values to that reported in the literature (Altringham & Young, 1991; Elliott *et al*. 2016). Time to peak twitch and half‐twitch relaxation time following supramaximal isometric twitch were measured as indicators of the rate of force development and the rate of muscle relaxation, respectively. Maximal isometric tetanic force at *L*_0_ was determined using a train of stimuli delivered at 200 Hz for 200 ms (defined as *P*_0_) (see Appendix, Fig. A1*C*). A 5 min rest period occurred after tetanus stimulation to allow for muscle recovery. Data were sampled at 10 kHz during isometric twitch kinetics. From these data, the twitch‐to‐tetanus ratio and the maximal isometric tetanic stress could be calculated.

#### Force–velocity characteristics

The force–velocity relationship of the muscle was determined using a series of after‐loaded isotonic contractions between ∼5% and 80% of *P*_0_. The force–velocity relationship was determined by fitting a hyperbolic linear function (Marsh & Bennett, 1986) to the data. The muscle's maximal shortening velocity at zero force (*V*_max_), expressed relative to muscle fibre length (*L*_0_), peak instantaneous isotonic power (${\dot{W}}_{\max}$) and the power ratio (${\dot{W}}_{\max}/P_{0}V_{\max}$), a measure of the curvature of the force–velocity relationship, were determined from the fitted force–velocity relationship.

#### Diaphragm power output during cyclic contractions

Following isometric muscle length optimization and isotonic data collection, the work loop technique (Josephson, 1985) was employed to quantify the mechanical power output of the hemidiaphragm section (Syme & Stevens, 1989; Altringham & Young, 1991) (see Appendix, Fig. A1*D–E*) during cyclical contractions. This process subjects hemidiaphragm muscles to cyclical length changes with phasic stimulation, incorporating periods of submaximal force generation during force development and relaxation, as well as variation in the velocity at which the muscle shortens and lengthens (Askew & Marsh, 1997; Kissane *et al*. 2018*a*). Consequently, this technique mimics the *in vivo* movement and activation of the diaphragm to assess its functional properties.

Muscles were subjected to a sinusoidal length trajectory with a strain amplitude of ±6.5% (Altringham & Young, 1991) of *L*_0‐F_ at six cycle frequencies ranging from 1–15 Hz) (Syme, 1990; Altringham & Young, 1991). At each cycle frequency, the train duration, strain and phase of stimulation were optimized to yield the maximal net power output, enabling us to determine performance in a standardized and comparative way. Data were sampled at 1000× cycle frequency. Five cycles were performed at each cycle frequency, with the data reported calculated as the mean of the middle three cycles. The range of cycle frequencies studied was selected based on the range over which the muscle could generate net positive work determined from preliminary measurements and our own past experience during work loop contractions with this muscle tissue. The cycle frequency range encompassed the breathing frequency of rats at rest and during exercise and was similar to that used in previous studies (Syme & Stevens, 1989; Syme, 1990; Altringham & Young, 1991; Stevens & Syme, 1993). The condition of the diaphragm preparation was monitored by performing a control 5 Hz work‐loop (train duration of 54 ms stimulations at a phase, relative to peak length, of −14 ms) every fourth set of cycles, and data were corrected for any decline in net power assuming a linear decline in muscle performance.

To examine the ability of the muscle to sustain net power output over repetitive activity, a fatigue run of 30 cycles at 2 Hz (train of 210 ms stimulations at a −20 ms phase) was performed. This standard condition was protocol across all muscles to represent typical frequencies of breathing *in vivo* (Syme & Stevens, 1989; Stevens & Syme, 1993). In this protocol, reductions in force, slowing of relaxation, and changes in the force–velocity relationship are accounted for by measuring net power (Askew *et al*. 1997; Tallis *et al*. 2014; Kissane *et al*. 2018*a*). Net power was normalized to the net power output of the first cycle. The mechanisms involved in power decline were subsequently derived from the fatigue test by calculating changes in the relaxation kinetics, determined from the work performed on the muscle during shortening and lengthening. Relative power has been graphically illustrated because each groups net power is partly related to the size of muscle preparation.

### Muscle composition, capillarity and oxygen modelling

#### Muscle fibre type composition and capillarity

Muscle segments from each hemidiaphragm were taken from the Krebs–Henseleit solution and mounted on to cork disks, snap frozen in isopentane cooled in liquid nitrogen and stored at −80°C. The muscle section which was used for the assessment of diaphragm physiological properties was used for immunohistological analysis (see Appendix, Fig. A1*F*). Serial sections of tissue (10 μm thick) were cut and tissue stored at −20°C until staining. Two monoclonal anti‐myosin heavy chain antibodies (Developmental Studies Hybridoma Bank, Iowa Cirty, IA, USA) were used to distinguish between muscle fibre types (Kissane *et al*. 2018*a*): BA‐D5 (1:1000) for Type I and SC‐71 (1:500) for Type IIa fibres. Any unstained fibres were determined to be Type IIx/IIb (Elliott *et al*. 2016). Muscle fibre boundaries were determined using an anti‐laminin staining (dilution 1:500; Cat# L9393; Sigma, St Louis, MO, USA) and capillaries with a carbohydrate‐binding protein (lectin), *Griffonia simplicifolia* lectin I (FL‐1101 GSL I; dilution 1:250; Vector Laboratories, Burlingame, CA, USA). Secondary antibodies were purchased from Thermo Fisher Scientific Inc. (Waltham, MA, USA) (dilution 1:1000; Alexa Fluor 488, Cat# A11001; Alexa Fluor 555, Cat# A31570). Laminin was biotinylated against anti‐rabbit IgG (Cat# BA1000; Vector Laboratories) and labelled with streptavidin (Cat# S11222; Pacific Blue Conjugate; Life Technologies, Carlsbad, CA, USA). Antibodies have been previously validated (Sonjak *et al*. 2019; Tickle *et al*. 2020). Imaging occurred at 20× magnification (Eclipse E600; Nikon, Tokyo, Japan) with three repeats per muscle section. Data were assessed for measures of both global [fibre type distribution (%), capillary‐to‐fibre (C:F) ratio, capillary density (mm^−2^) and fibre type cross‐sectional area (μm^2^)] and local [capillary domain area (μm^−2^; defined as tessellating Voronoi polygons); local capillary to fibre type ratio (LCFR); and local capillary density (LCD) (mm^−2^)] morphometric indices calculated using Dtect and oxygen transport modelling software (Kissane *et al*. 2018*a*,*b*; Al‐Shammari *et al*. 2019). The Dtect and oxygen transport modelling software was developed by the authors presented in (Al‐Shammari *et al*. 2019) and was specifically written for the analysis and oxygen modelling of tissue based on the true anatomical structure of the specific muscle, unlike other available alternatives, which use theoretical structures. As such, the Dtect and oxygen transport modelling software can produce a physiologically relevant data set. Data are presented as raw values for each group, and in the ipsilateral (paralysed) hemidiaphragm relative to the amount in the corresponding contralateral hemidiaphragm. Data are presented in the normalized form to the contralateral hemidiaphragm of the same animal avoid effects of inter‐animal variability.

Microvascular composition is most commonly described through gross, global, scale‐dependent integers. Use of the Dtect system allows us to determine assessments of local capillary distribution within the diaphragm tissue, providing data with substantial analytical resolution concerning microvascular remodelling (Egginton & Gaffney, 2010). Although a large number of local capillary indices exist (Egginton, 1990), those most commonly utilized are insensitive to changes during tissue remodelling (Olfert *et al*. 2016), as occurs within the diaphragm following injury induced paralysis.

#### Oxygen tension modelling

Using oxygen transport modelling software (Al‐Shammari *et al*. 2019), we generated theoretical predictions of oxygen tension cross‐sectional distribution within muscle tissue. To date, this oxygen model transport modelling software utilizes the most biophysically appropriate techniques to predict oxygen tension across histological derived morphometrics. Default parameters were used with control tissues and values for the maximal rate of oxygen consumption ($M_{O_{2}\max}$) were subsequently changed for groups 3 and 4 based upon the results of the citrate synthase activity (giving an indication of intact mitochondria). Exercise level for all samples was set to moderate and the level of differential extraction of oxygen was low. Data were assessed for simulated measures of average partial pressure of oxygen in the tissue ($P_{O_{2}}$) and the oxygen consumption of the tissue ($M_{O_{2}}$).

#### Citrate synthase assay for mitochondrial activity

The muscle samples caudal to those used for physiological assessment and IHC were snap frozen and stored at −80°C until assessment. The samples were thawed and then ∼15 mg of muscle was homogenized in 1 ml of buffer (100 mm sucrose, 50 mm KCl, 5 mm EDTA, 50 mm Tris‐base and pH 7.4). The homogenate underwent two freezing/thaw cycles then centrifuged (1000 *g* for 10 min). The supernatants were used for the following enzyme assays. Assays were conducted at 37°C and assessed in a reaction medium [100 mm Tris buffer, pH 8; 100 μm 5,5‐dithiobis (2‐nitrobenzoic acid); 300 μm acetyl‐CoA] with 70 μg ml^−1^ of supernatant. Following 3 min of incubation, enzyme activity was initiated by addition of 500 μm oxaloacetate. The reaction (a reduction of DNTB by CoASH) was assessed on a multiplate reader (Thermo Scientific, Waltham, MA, USA) through a change in optical density at 412 nm.

### Statistical analysis

The incidence of spontaneous SCI is negligible in the laboratory rat population. As such, power analysis using G*Power (https://www.gpower.hhu.de) was conducted prior to all experiments to ensure sample sizes used were sufficient to yield reliable data based on previous experimental data to determine the expected effect size, level of acceptable significance and type 1 error threshold (α) ≤ 0.05 and power (1 – β) ≥ 0.90). The functional, histological and behavioural recordings from every animal in each group were analysed without exclusion based on outcome. Statistical analyses were performed using one‐way or two‐way ANOVA and *post hoc* comparisons were conducted using the Bonferroni correction or, for non‐parametric tests, the Kruskal–Wallis test with *post hoc* Dunn's for multiple comparisons assessment. All statistical assessments were performed using GraphPad Prism (GraphPad Software Inc., San Diego, CA, USA). Divergences were considered significant if *P* < 0.05. Data are presented as the mean ± SD.

## Results

### Induced spinal plasticity restores diaphragm motor function

Uninjured animals (group 1, sham injured control) (see Appendix, Fig. A1*A*) showed strong and synchronized diaphragm motor function during eucapnic conditions through EMG recordings of bilateral hemidiaphragm function 6 weeks following surgery (Fig. 1 *A* and *D*). However, upon receiving the lateral cervical (C) level 2 hemisection (see Appendix, Fig. A1*B*; groups 2–4), all animals lost complete function in the ipsilateral hemidiaphragm (shown at 6 weeks post injury for groups 2&3 and pre‐treatment at 4 weeks post injury for group 4). This extensive injury caused physiologically complete hemidiaphragm paralysis, which persisted in all animals throughout the course of our experiment or until treatment was applied (Fig. 1 *B–D*). The paralysis was a result of the severing of descending serotonergic, glutamatergic and pre‐phrenic interneuronal and motor pathways that ultimately innervate the PMP (see Appendix, Fig. A1*B*) (Porter, 1895). Importantly, the contralateral side of the cord is not injured and, subsequently, the contralateral hemidiaphragm functions to maintain the respiratory and ventilatory parameters of the animal. Producing an extensive high cervical hemisection that results in chronic paralysis is infrequent within the literature (Nantwi *et al*. 1999; Golder & Mitchell, 2005; Lane *et al*. 2008*a*) but important for the present study because it enables us to accurately assess the effects of injury and recovery on muscle function and morphology (Warren *et al*. 2018*b*). Frequency, breath and cycle length (Fig. 1 *E–G*) were unchanged following injury during eupnic breathing.

Treatment was applied to animals in group 4 (see Appendix, Fig. A1*A*; ChABC animals) 4 weeks following the C2 hemisection through a single injection of ChABC into the ipsilateral C4 PMP. The site of treatment application was critical, targeting sprouting and recovery at the site of diaphragm spinal innervation and not through the injury site itself. ChABC restored synchronized diaphragm function in all of animals 2 weeks following application (ANOVA, *P* < 0.0001, *F*_3,25_ = 66.99; 6 weeks following the initial SCI) (Fig. 1 *C–D*). The degree of recovery was ∼50% of that achieved during respiratory challenge by airway occlusion and was not substantially different from results obtained from the contralateral hemidiaphragm (Fig. 1 *D*). This was a slight deficit but not statistically divergent to the ∼60% maximal ipsilateral activity shown by control animals (Fig. 1 *D*) (Mantilla *et al*. 2010). Activity in the contralateral hemidiaphragm was unchanged regardless of experimental group (ANOVA, *P* = 0.148, *F*_3,25_ = 1.95). Restoration of function following ChABC treatment caused an increase in breath duration (ANOVA, *P* = 0.003, *F*_3,25_ = 6.20) with a corresponding decrease in cycle length (ANOVA, *P* = 0.008, *F*_3,25_ = 4.93), resulting in a constant breath frequency (ANOVA, *P* = 1.28, *F*_3,25_ = 2.09) compared to control and injured animals (Fig. 1 *E–G*). This is consistent with a need to increase blood oxygen levels in the recovering animal.

To investigate the capacity of ChABC to elicit recovery at different stages post injury, we assessed the application of the enzyme at acute to chronic time points following trauma. These data included animals separate to that reported previously. Our data showed that ChABC treatment at the level of the PMP applied at acute stages (0–7 days) following C2 hemisection caused meagre, if any, recovery of inspiratory function when assessed two weeks later (Fig. 1 *H*). However, application of the enzyme at chronic time points (4 and 12 weeks following injury) resulted in an increasingly more robust, and synchronized recovery of ipsilateral diaEMG activity. These results show that induction of plasticity and CSPG removal at areas of respiratory innervation can recover substantial motor function after injury, and that this method of inducing recovery works better when applied at chronic time points rather than acutely.

### Induced neurological plasticity can restore diaphragm functional properties

**Figure 2:**
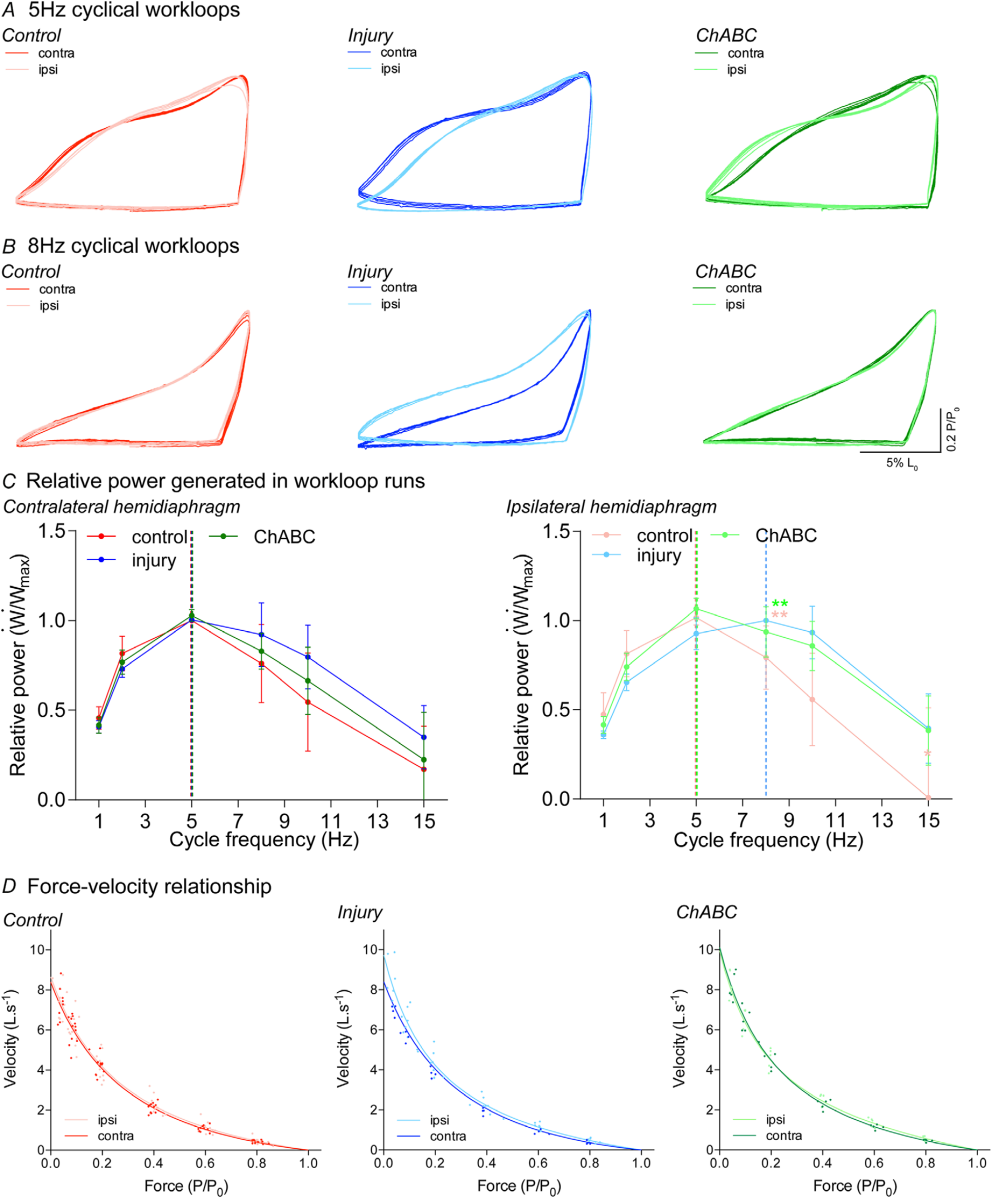
Injury alters optimal diaphragm functional parameters, which are shifted back towards normal following ChABC treatment *A* and *B*, representative work loops at 5 Hz (*A*) and 8 Hz (*B*). In all muscles there is little change in re‐lengthening. *C*, relative net power–frequency relationship (normalized to the maximum net power) for the contralateral and ipsilateral hemidiaphragms. Vertical dashed lines indicate the cycle frequency at which maximum relative power is generated from the different experimental groups. Data assessed via Kruskal–Wallis with the following sample sizes: control ipsi = 14; control contra = 10; injury ipsi = 7; injury contra = 6; ChABC ipsi = 5; ChABC contra = 5. *D*, force–velocity relationship for all groups. All data are from control (red; group 1), injury (blue; groups 2&3) and ChABC (green; group 4) animals. ^*^ *P* < 0.05 and ^**^ *P* < 0.01. If no *post hoc* result is shown, the comparison was not significant. Values represent the mean ± SD. Abbreviations: Ipsi, ipsilateral; contra, contralateral hemidiaphragm recordings. [Color figure can be viewed at wileyonlinelibrary.com]

To determine the mechanism behind the swift recovery in muscle activity, we evaluated functional properties of the diaphragm following both injury and ChABC‐mediated functional recovery. This (and all subsequent experiments) was performed on animals 6 weeks following injury (described in groups 1–4). Assessment of hemidiaphragm properties using traditional isometric and isotonic measures (associated with either twitch or tetanus contractions; see Appendix, Fig. A1*C*) yielded only modest alterations in response to injury induced paralysis and ChABC‐mediated recovery of function (see Appendix, Tables A1 and A2) (Miyata *et al*. 1995; Ameredes *et al*. 2000; Mantilla *et al*. 2013*b*; Elliott *et al*. 2016). However, assessment of diaphragm performance using these traditional techniques is limited because they do not mimic how the muscle functions *in vivo*. The resulting data therefore do not accurately reflect the muscles functional properties. To provide a more clinically relevant and accurate assessment of muscle performance, which mimics the cyclical length changes and phasic activation *in vivo* movement and a more realistic activation pattern of the diaphragm, we measured muscle function in all experimental groups during repeated cycles of contraction using the innovative work loop technique (Fig. 2 *A* and *B*) (Josephson, 1985).

**Figure A2:**
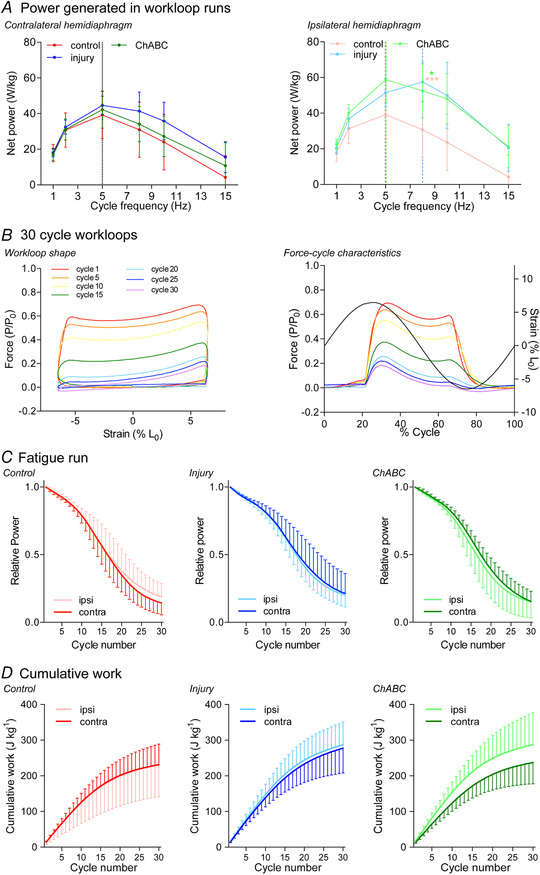
Effect of numerous fatigue cycles on the work produced by the diaphragm *A*, power–frequency relationship for the contralateral and ipsilateral hemidiaphragms. Vertical dashed lines represent optimal frequency from different experimental groups. Data assessed via Kruskal‐Wallis with the following sample sizes: control ipsi = 12; control contra = 10; injury ipsi = 6; injury contra = 6; ChABC ipsi = 6; ChABC contra = 5. *B*, representative examples of 30 continuous 2 Hz fatigue cycles on work‐loop shape and force–cycle characteristics (from ipsilateral control tissue) demonstrating how the run reduces the net power output of the muscle. *C*, relative net power output during a 2 Hz fatigue run with power relative to the initial cycle. All graphs show control (red), injury (blue) and ChABC (green) groups. Data assessed via two‐way ANOVA with the following sample sizes: control ipsi = 12; control contra = 10; injury ipsi = 7; injury contra = 6; ChABC ipsi = 5; ChABC contra = 5. *D*, cumulative work produced during a 2 Hz fatigue run. Data assessed via two‐way ANOVA with the following sample sizes: control ipsi = 12; control contra = 10; injury ipsi = 7; injury contra = 6; ChABC ipsi = 5; ChABC contra = 5. Graphs show control (red; group 1), injury (blue; groups 2&3) and ChABC (green; group 4) animals. ^*^ *P* < 0.05 and ^***^ *P* < 0.001. If no *post hoc* result is shown, the comparison was not significant. Values represent the mean ± SD. Abbreviations: Ipsi, ipsilateral; contra, contralateral hemidiaphragm recordings. [Color figure can be viewed at wileyonlinelibrary.com]

The relationships between relative net power produced by the muscle and cycle frequency of the contralateral hemidiaphragms and ipsilateral controls were similar (Fig. 2 *C*). These data show that the optimal frequency for maximum relative net power generation of a normal functioning diaphragm muscle occurs at a cycle frequency of 5 Hz (Fig. 2 *C*; see also Appendix, Fig. A2*A*) (Altringham & Young, 1991; Stevens & Syme, 1993). This represents the breathing frequency at which the rat could generate the most net work and net power, and is similar to the breathing frequency used during moderately intense activity (Altringham & Young, 1991). Representative work loops of optimal frequency of power generation for the different groups (Fig. 2 *A* and *B*) are indicated at the point of the dashed line in Fig. 2 *C*.

Following SCI‐induced diaphragm paralysis, there was a significant shift in the optimal cycle frequency for maximum relative net power of the ipsilateral hemidiaphragm from 5 Hz to 8 Hz (Kruskal–Wallis, *H*_6_ = 22.05, *P* = 0.0005, *post hoc* Dunn's *P* > 0.01) (Fig. 2 *A–C*). This corresponded to an increase in relative net power generated at the higher cycle frequencies (Kruskal–Wallis, *H*_6_ = 20.2, *P* = 0.0011) (see Appendix, Fig. A2*A*). This is consistent with alterations in the force–velocity relationship of the muscle (Fig. 2 *D*; see also Appendix, Table A2). The paralysed hemidiaphragm muscle was able to produce more force at higher cycle frequencies, which would occur during intense activity (Fig. 2 *A–C*), but less relative power at the lower frequencies typical of breathing at rest (Fig. 2 *C*). As such, SCI induced paralysis caused the ipsilateral hemidiaphragm to no longer be optimized for low to moderate breathing. ChABC‐mediated partial restoration of neurological input and activity caused a significant shift in the optimal cycle frequency for the ipsilateral hemidiaphragm back to control levels (5 Hz; *post hoc* Dunn's *P* > 0.01) (Fig. 2 *A–C*). This recovery in muscle functional properties facilitated the rapid normalization of diaphragm activity demonstrated within the EMG recordings (Fig. 1 *C–D*). Although recovery within the ipsilateral hemidiaphragm within the treated group resolved optimum frequency, there were increases of power at higher frequencies compared to control. No animal could reasonably breath at these rates, and it is an indication that the recovery is in transition and not fully complete, as was also demonstrated in EMG recordings (Fig. 1).

Deficits in a muscles capacity to contract, and thus generate and sustain force, can lead to increased susceptibility to fatigue. We showed a significant main effect between all muscles over the course of the fatigue run (two‐way ANOVA, injury: *P* < 0.0001, *F*_5,1170_ = 7.421; cycle number: *P* < 0.0001, *F*_29,1170_ = 335.5) (see Appendix, Fig. A2*C*). This is probably caused by the higher initial power generating capacity of the ipsilateral hemidiaphragm following injury and ChABC‐mediated recovery of function at this frequency, as well as alterations in cumulative work performed by the muscle (see Appendix, Fig. A2*A* and *D*). This confirms modest alteration in fatigue resistance of the diaphragm following muscle paralysis and subsequent ChABC‐mediated recovery (Stevens & Syme, 1993).

### Recovery of activity following paralysis causes restoration in diaphragm global morphology

Modifications to the hemidiaphragms phenotype, driven by alterations in vascular supply and metabolic imbalance, could explain why the functional dynamics of the ipsilateral hemidiaphragm change in response to paralysis and how the muscle is able to retain some capacity to function (although not optimally). To address this, we assessed global measures of tissue morphology and capillary supply using the muscle sections isolated for muscle kinetics. Because the diaphragm shows a heterogeneous fibre type distribution across the costal region (thoracic and abdominal sides; see Appendix, Fig. A1*D*) (Metzger *et al*. 1985), the data generated are representative of each hemidiaphragm as a whole.

**Figure 3:**
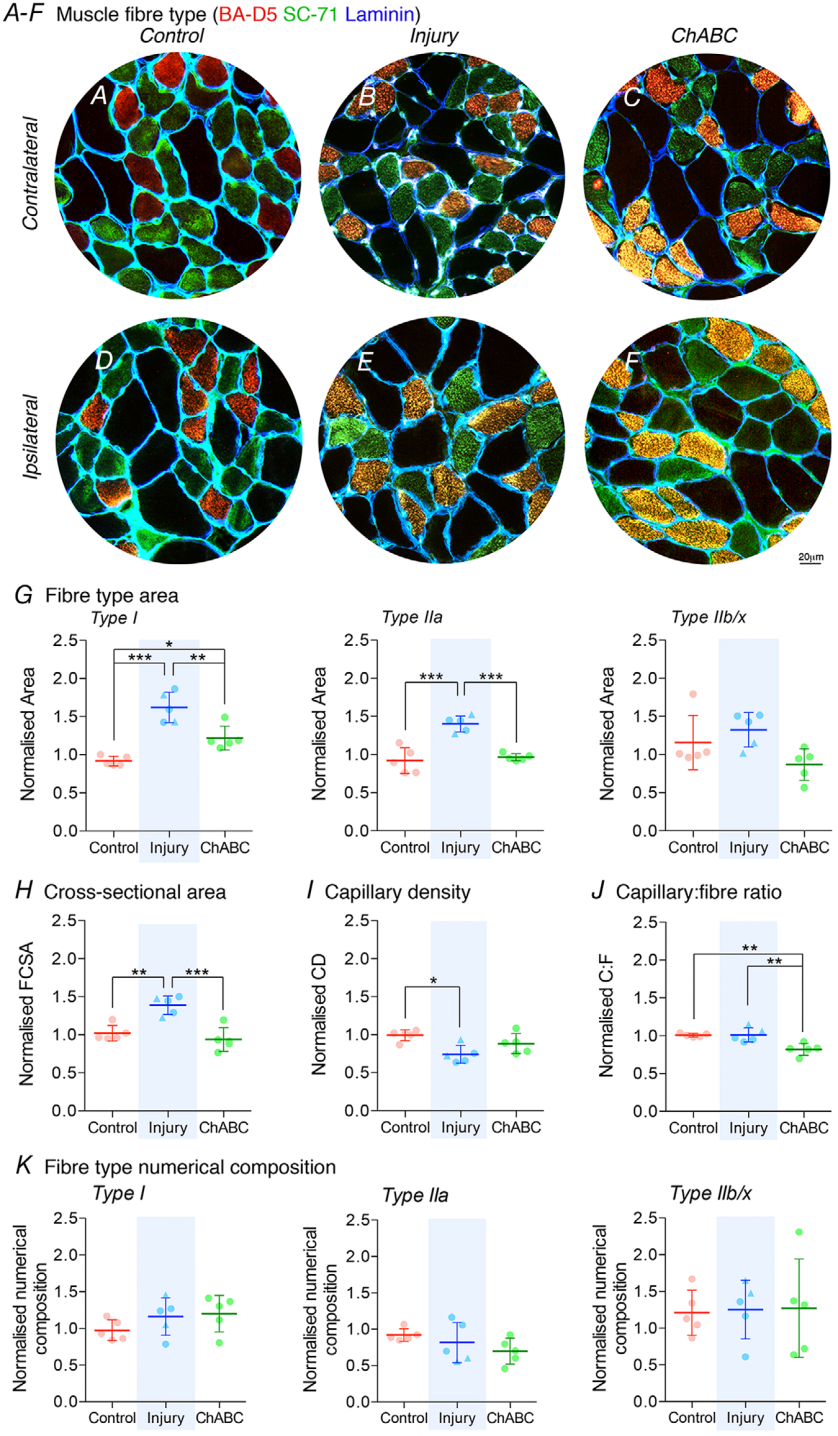
Injury causes hypertrophy of ipsilateral muscle fibres, which is resolved following ChABC treatment and recovery of EMG function *A*–*F*, immunohistochemistry of diaphragm muscle for Type I (BA‐D5; red), Type IIa (SC‐71; green) and laminin (blue). *G*, fibre type specific changes for average fibre area. The global angiogenic response to injury and ChABC treatment shown through (*H*) fibre cross‐sectional area (FCSA), (*I*) capillary density (CD) and (*J*) the capillary to fibre ratio (C:F). *K*, fibre type specific changes for relative numerical density. All graphs show control (red; group 1), injury (blue; groups 2&3) and ChABC (green; group 4) animals. The data presented are normalized (ipsilateral/contralateral results from the same animal) to control for variance of all extenuating factors. Raw data are presented in the Appendix (Fig. A3). All assessed via ANOVA with all groups of sample size = 5. ^*^ *P* < 0.05, ^**^ *P* < 0.01 and ^***^ *P* < 0.001. If no *post hoc* result is shown, the comparison was not significant. Scale bar = 20 μm. Values represent the mean ± SD. Triangle data points represent group 3 animals. [Color figure can be viewed at wileyonlinelibrary.com]

**Figure A3:**
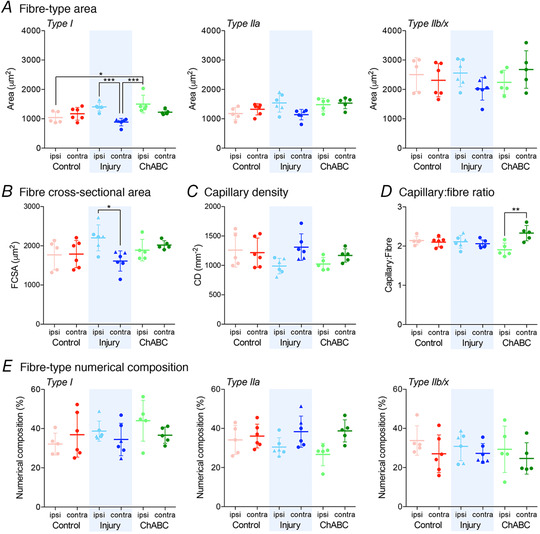
Raw data from experimental groups showing the hypertrophy of muscle fibres caused by the injury and subsequent rarefaction generated by ChABC mediated recovery of function *A*, fibre type specific changes for average fibre area. The global angiogenic response to injury and ChABC treatment shown through (*B*) fibre cross‐sectional area (FCSA), (*C*) capillary density (CD) and (*D*) the capillary to fibre ratio (Capillary:Fibre). *E*, fibre type specific changes for relative numerical density. All graphs show control (red; group 1), injury (blue; groups 2&3) and ChABC (green; group 4) animals. Normalized data shown in Figure 3. Data assessed via ANOVA with sample sizes: control ipsi = 5; control contra = 6; injury ipsi = 6; injury contra = 6; ChABC ipsi = 5; ChABC contra = 5. ^*^ *P* < 0.05, ^**^ *P* < 0.01 and ^***^ *P* < 0.001. If no *post hoc* result is shown, the comparison was not significant. Values represent the mean ± SD, *n* = 5–7 per group. Triangle data points represent group 3 animals. Abbreviations: Ipsi, ipsilateral; contra, contralateral hemidiaphragm recordings. [Color figure can be viewed at wileyonlinelibrary.com]

Control animals showed no significant difference between the ipsilateral and contralateral sections of the hemidiaphragm for any global morphological measure assessed (Fig. 3; see also Appendix, Fig. A3). However, chronic paralysis did cause alterations in the ipsilateral hemidiaphragm following injury. Type I and IIa fibres were significantly larger in this muscle (ANOVA, Type I: *P* < 0.0001, *F*_2,12_ = 27.3, Type IIa: *P* < 0.0001, *F*_2,12_ = 25.7) (Fig. 3 *A–G*; see also Appendix, Fig. A3*A*). A result confirmed through assessment of the mean fibre cross‐sectional area (ANOVA, *P* = 0.0003, *F*_2,12_ = 17.3) (Fig. 3 *B*; see also Appendix, Fig. A3*B*). This hypertrophic response would enable the individual muscle fibres to generate more force, explaining how the paralysed muscle is still able to generate substantial power (see Appendix, Fig. A2*A*). However, the expansion in fibre area increased diffusion distances for fuel (oxygen and glucose) and carbon dioxide to the fibre core. This can be seen through a decrease in the ipsilateral hemidiaphram capillary density following chronic injury induced paralysis (ANOVA, *P* = 0.012, *F*_2,12_ = 6.51) (Fig. 3 *I*; see also Appendix, Fig. A3*C*). This effect was caused by an increase in fibre size rather than change in capillary number (Fig. 3 *J*). These alterations in diffusion distances for fuel and waste products within the chronically paralysed muscle would facilitate a shift in hemidiaphragm optimal functional kinetics. An increase in size of contralateral Type I and IIa fibres following injury indicates a greater requirement for fuel in this tissue, which is now having to perform more work per fibre to maintain respiratory function (see Appendix, Fig. A3*A*).

The functional activity and properties of the ipsilateral hemidiaphragm were largely restored following ChABC treatment at chronic time points (Fig. 1 *C–D* and 2 *C*). Uniquely, we show that this recovery in diaphragm function occurred with a simultaneous restoration of global indices of muscle phenotype. The size of Type I and IIa fibres tended to return to control values following ChABC‐mediated recovery (Fig. 3 *A–G*; see also Appendix, Fig. A3*A*), although Type IIb/x fibre areas were largely unaffected (ANOVA, *P* = 0.0582, *F*_2,12_ = 3.64). This was echoed in a restoration of the muscles mean fibre cross‐sectional area (Fig. 3 *H*). This reduction in fibre size, particularly of the slow fibres most used by the diaphragm during normal breathing, explains why the power produced by this muscle tends to revert to optimal control values following ChABC treatment (Fig. 2 *C*). The normalization of capillary density within the ipsilateral hemidiaphragm following recovery of activity (Fig. 3 *I*) is partly caused by the reduction in size of the muscle fibres. However, it was also triggered by an angiogenic response in the contralateral hemidiaphragm (ANOVA, *P* = 0.0017, *F*_2,12_ = 11.4) (Fig. 3 *J*), evidenced by a significant increase in the capillary‐to‐fibre ratio of this muscle (see Appendix, Fig. A3*D*).

Importantly, the numerical fibre type composition of each hemidiaphragm remained constant (∼30–40% total area) regardless of the experimental condition (ANOVA, Type I: *P* = 0.268, *F*_2,12_ = 1.47, Type IIa: *P* = 0.244, *F*_2,12_ = 1.59, Type IIb/x: *P* = 0.979, *F*_2,12_ = 0.021) (Fig. 3 *A–F* and *K*; see also Appendix, Fig. A3*E*). This is consistent with the modest changes we observed in twitch‐tetanus kinetics (see Appendix, Tables A1 and A2) and the force–velocity relationship (Fig. 2 *D*) (Metzger *et al*. 1985; Miyata *et al*. 1995; Sieck *et al*. 2012; Mantilla *et al*. 2013*b*). This uniformity in fibre type composition explains the rapid restoration of typical respiratory activity once the muscle is reinnervated. Because the fibres have not shifted to a faster phenotype following paralysis, normal breathing at rest (which utilizes slow muscle fibres) can readily occur once activity resumes within the motor system.

### Restoration of diaphragm function is driven by local vascular and oxygen supply in the muscle

**Figure 4:**
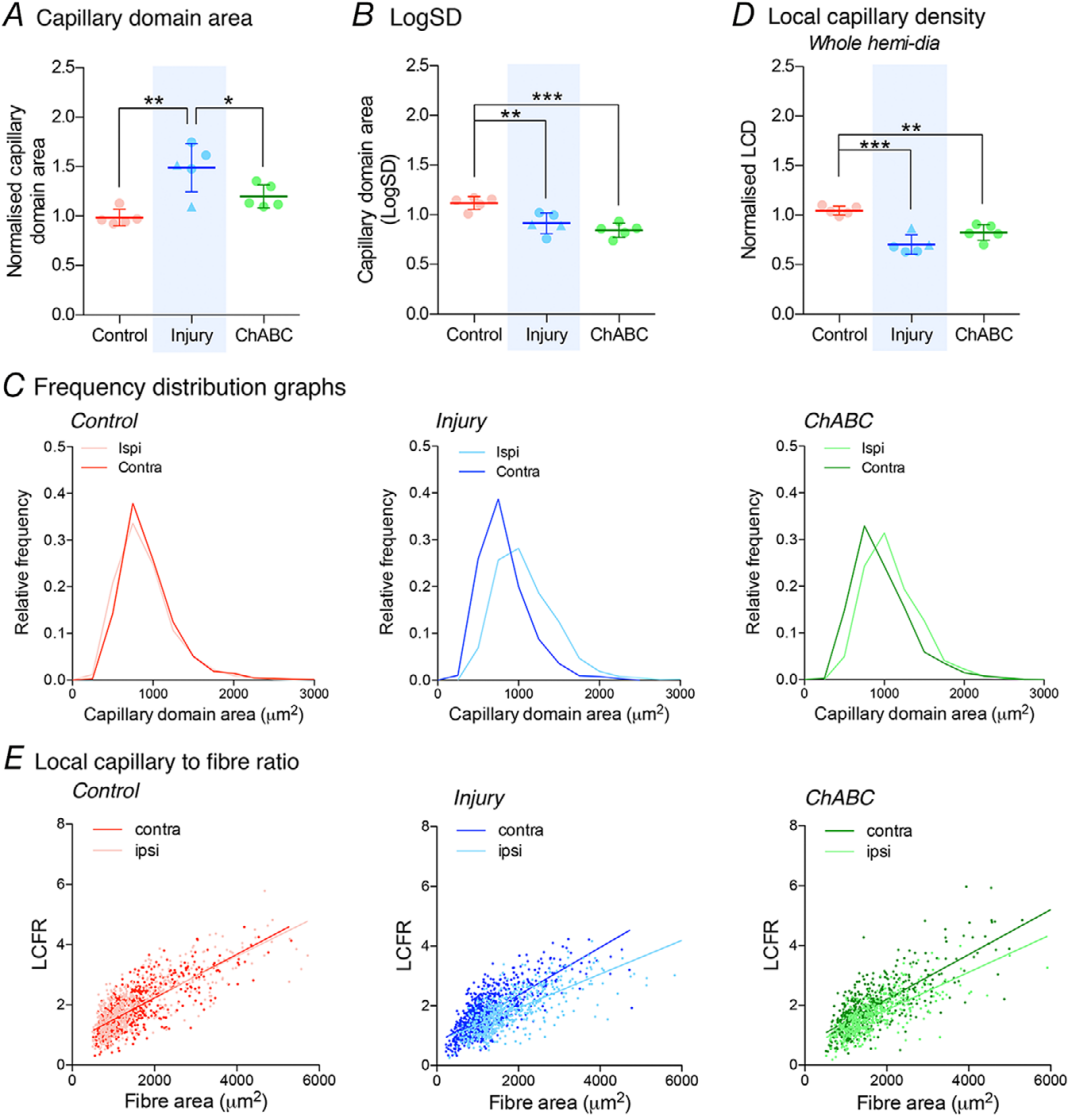
Local changes in microvascular composition facilitate supply for oxygen in diaphragm tissue after spinal injury Capillary domain area (*A*), capillary heterogeneity presented as LogSD (*B*) and frequency distribution for the capillary domain areas (*C*). The data presented are normalized (ipsilateral/contralateral results from the same animal) to control for variance of all extenuating factors. Local capillary supply shown for the whole hemidiaphragm through (*D*) local capillary density and (*E*) local capillary to fibre ratio shown correlated for fibre size. All graphs show control (red; group 1), injury (blue; groups 2&3) and ChABC (green; group 4) animals. Additional normalized and raw data shown in the Appendix (Fig. A4). All assessed via ANOVA with all groups of sample size = 5. ^*^ *P* < 0.05, ^**^ *P* < 0.01 and ^***^ *P* < 0.001. Values represent the mean ± SD. Triangle data points represent group 3 animals. Abbreviations: Ipsi, ipsilateral; contra, contralateral hemidiaphragm recordings. [Color figure can be viewed at wileyonlinelibrary.com]

**Figure A4:**
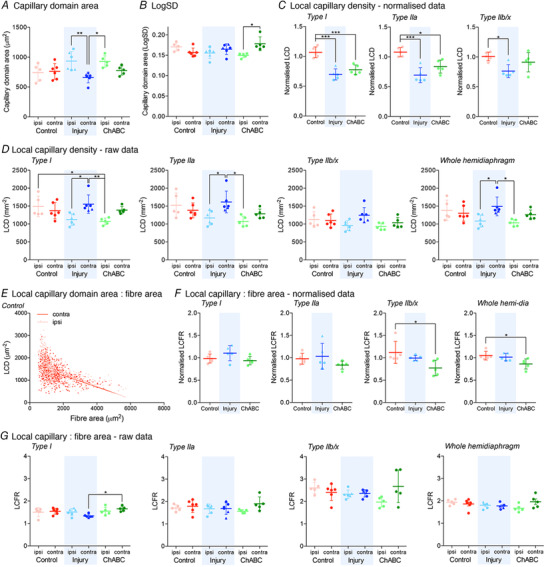
Raw and normalized data from experimental groups showing the local changes in angiogenesis caused by the injury Capillary domain area (*A*) and capillary heterogeneity (*B*) presented as LogSD. Normalized (*C*) and raw (*D*) local capillary supply density shown for each fibre type. *E*, local capillary supply density correlated for fibre size for control tissue. Normalized (*F*) and (*G*) raw local capillary to fibre ratio shown for each fibre type and whole diaphragm. All graphs show control (red; group 1), injury (blue; groups 2&3) and ChABC (green; group 4) animals. Additional and normalized data shown in Fig. 4. Data presented in (*C*) and (*F*) are normalized (ipsilateral/contralateral results from the same animal) to control for variance of all extenuating factors. Data assessed via ANOVA with normalized sample size of groups = 5. Sample sizes raw data: control ipsi = 5; control contra = 6; injury ipsi = 6; injury contra = 6; ChABC ipsi = 5; ChABC contra = 5. ^*^ *P* < 0.05, ^**^ *P* < 0.01 and ^***^ *P* < 0.001. If no *post hoc* result is shown, the comparison was not significant. Values represent the mean ± SD. Triangle data points represent group 3 animals. Abbreviations: Ipsi, ipsilateral; contra, contralateral hemidiaphragm recordings. [Color figure can be viewed at wileyonlinelibrary.com]

Our data have shown increased spatial distribution of microvascular supply within hemidiaphragm tissue following SCI. However, gross, global assessments of muscle morphology are incapable of fully describing a dynamic response within the muscle microvasculature to paralysis or disease (Egginton & Gaffney, 2010). Local, vascular adaptions to injury are based on the quantification of capillary domains (tessellated areas within a tissue that predict supply regions for an individual capillary). Control animals showed no significant difference between ipsilateral and contralateral sections of the hemidiaphragm for all local capillary indices assessed (Fig. 4; see also Appendix, Fig. A4). Conversely, chronic paralysis of the ipsilateral hemidiaphragm caused pronounced differences in these measures. The median capillary domain area of the ipsilateral muscle significantly increased (ANOVA, *P* = 0.0014, *F*_2,12_ = 12.0) (Fig. 4 *A*; see also Appendix, Fig. A4*A*). This is a result of muscle fibre hypertrophy, meaning that the area of tissue each capillary has to supply with fuel is increased. However, the increase in oxidative muscle fibre size simultaneously decreased the spatial heterogeneity (LogSD) of capillary domain areas in the ipsilateral muscle following injury‐induced muscle paralysis (ANOVA, *P* = 0.0005, *F*_2,12_ = 15.0) (Fig. 4 *B*; see also Appendix, Fig. A4*B*). Indeed, chronic paralysis of the ipsilateral hemidiaphragm caused a rightward shift in capillary domain frequency distribution (Fig. 4 *C*), characteristic of muscle hypertrophy, with a simultaneous increase in the median capillary domain area. To assess this further, we evaluated the degree to which local capillary supply of the diaphragm was modified following chronic injury‐induced paralysis. The LCD of the muscle is a scale‐independent measure and thus a more accurate assessment of the functional vascular supply. Our data showed that capillarization of the muscle significantly decreased following injury‐induced paralysis of the ipsilateral hemidiaphragm (ANOVA, *P* < 0.0001, *F*_2,12_ = 24.9) (Fig. 4 *D*; see also Appendix, Fig. A4*C*). This was evident for all fibre types (see Appendix, Fig. A4*C–E*), although it was particularly striking with respect to oxidative fibres. This would negatively impact the functional capacity of the hemidiaphragm, corroborating the shift in optimal functional properties exhibited by the muscle (Fig. 2 *C*). As a result of these alterations in spatial distribution of capillaries within the hemidiaphragm, we assessed the microvascular transport capacity to the muscle fibres (LCFR; the sum of the fractional domain area overlapping each fibre). We demonstrated a significant trend for deficits within this measure following injury‐induced paralysis of the ipsilateral hemidiaphragm, particularly affecting the larger fibre types (ANOVA, *P* = 0.0254, *F*_2,12_ = 5.06) (Fig. 4 *E*; see also Appendix, Fig. A4*E–G*). This reduction in transport capacity would have facilitated modification to the muscles functional properties observed following injury (Fig. 2 *C*).

Recovery of function in the ipsilateral hemidiaphragm following ChABC treatment occurred concurrently with alterations in the local morphology of the muscle. The reduction in ipsilateral muscle fibre size decreased the mean capillary domain area within the muscle (Fig. 4 *A*; see also Appendix, Fig. A4*A*). However, a simultaneous angiogenic response in the contralateral hemidiaphragm resulted in further decreases in the spatial heterogeneity of capillary domains (Fig. 4 *B*). These data are supported by changes in capillary domain frequency distribution (Fig. 4 *C*), showing the relative frequency of domain areas of the ipsilateral and contralateral hemidiaphragm equalize following ChABC‐mediated diaphragm recovery, although they do not return to control levels. However, the median frequency of the domains shifted to the left, reflecting a reduced supply area for individual capillaries. Critically, this novel assessment of local muscle morphology has identified key changes in the diaphragm tissue after neurological recovery which will facilitate improved oxygen transport within the tissue. However, these alterations do not represent a complete restoration of the morphology to pre‐injury conditions. To assess this further, we evaluated capillarization of the diaphragm following ChABC‐induced respiratory motor recovery. The local capillary density of the diaphragm increased towards control values (Fig. 4 *D*) although this was largely a result of decreases in the capillary density of the contralateral hemidiaphragm in all fibre types (see Appendix, Fig. A4*C–D*). Although not identical to control values, normalizing these values would facilitate a shift of muscle functional properties to optimal levels (Fig. 2 *C*), at the same time as mediating a divergence in power produced at the higher frequencies following ChABC‐mediated recovery (see Appendix, Fig. A2*A*). Indeed, these alterations increased microvascular transport capacity of the contralateral hemidiaphragm for both fast oxidative and glycolytic muscle fibres (ANOVA, *P* = 0.0254, *F*_2,12_ = 5.06) (Fig. 4 *E*; see also Appendix, Fig. A4*F–G*), demonstrating an increased capacity for supply of oxygen and other fuels in the muscle by diffusion. The critical alterations in capillary distribution within the hemidiaphragm at both global and local levels following SCI‐induced paralysis (and the adaptions shown to normalize values following ChABC‐mediated respiratory recovery) suggests that oxidative potential within the muscle underpins the functional capacity and rapid restoration of activity within the diaphragm.

### Respiratory recovery rescues diaphragm oxygen supply but not the demand

**Figure 5:**
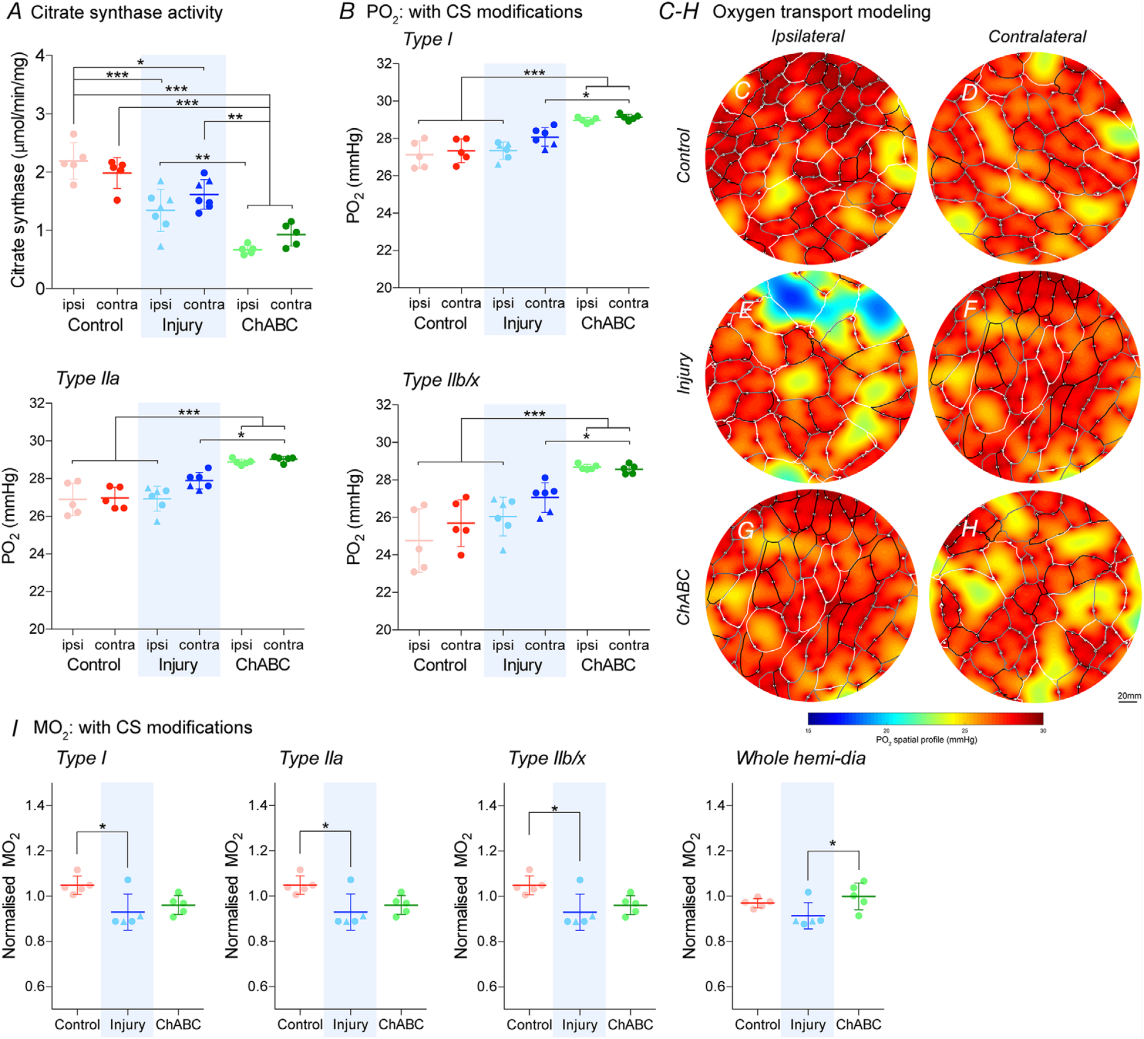
Demand for oxygen deficient the diaphragm following both spinal injury and neurological recovery *A*, amounts of citrate synthase activity in hemidiaphragm muscle tissue. Sample sizes: control ipsi = 5; control contra = 5; injury ipsi = 7; injury contra = 7; ChABC ipsi = 5; ChABC contra = 5. *B*, oxygen transport models for moderate muscle activity showing ${P_{o}}_{{}_{2}}$ of diaphragm tissue with alterations for mitochondrial content (CS). *C*–*H*, representative images of oxygen transport modelling showing a ${P_{o}}_{{}_{2}}$ of 15 mmHg (blue) to 30 mmHg (red). *I*, Oxygen transport models for moderate muscle activity showing $M_{O_{2}}$ of diaphragm tissue with CS alterations. The data presented are normalized (ipsilateral/contralateral results from the same animal) to control for variance of all extenuating factors. All graphs show control (red; group 1), injury (blue; groups 2&3) and ChABC (green; group 4) animals. Additional normalized and raw data shown in the Appendix (Fig. A5). Data assessed via ANOVA. *B* and *I*, normalized sample size of groups = 5. Sample sizes raw data: control ipsi = 5; control contra = 5; injury ipsi = 6; injury contra = 6; ChABC ipsi = 5; ChABC contra = 5. ^*^ *P* < 0.05, ^**^ *P* < 0.01 and ^***^ *P* < 0.001. If no *post hoc* result is shown, the comparison was not significant. Scale bar = 20 μm. Values represent the mean ± SD. Triangle data points represent group 3 animals. Abbreviations: Ipsi, ipsilateral; contra, contralateral hemidiaphragm recordings. [Color figure can be viewed at wileyonlinelibrary.com]

To determine the effect that global and local alteration in diaphragm morphology have upon diffusion dynamics within the tissue, we modelled oxygen transport within the muscle. This approach allows us to investigate the complex interaction between muscle supply and demand in response to dynamic remodelling of the muscle tissue and microvasculature after injury‐induced chronic paralysis and ChABC‐mediated restoration of activity (Fig. 5). Based on normal diaphragm activity and parameters of muscle composition, we simulated the tissue's average partial pressure of oxygen ($P_{O_{2}}$; correlate of tissue oxygen supply) and muscle oxygen uptake ($M_{O_{2}}$; correlate of tissue oxygen demand). Utilizing the citrate synthase assay (ANOVA, *P* < 0.0001, *F*_5,28_ = 24.4) (Fig. 5 *A*), we incorporated tissue‐derived values of mitochondrial content into our model to increase accuracy of the data obtained.

**Figure A5:**
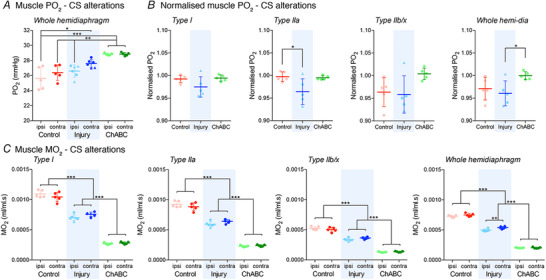
Raw and normalized data for $P_{O_{2}}$ and $M_{O_{2}}$ following diaphragm oxygen transport modelling Oxygen transport models for moderate muscle activity showing (*A* and *B*) raw and normalized $P_{O_{2}}$ and (*C*) raw $M_{O_{2}}$ for all fibre types and the whole hemidiaphragm. Modelling occurred following adjusting tissue $M_{O_{2}}$. values according to mitochondrial content (CS). All graphs show control (red; group 1), injury (blue; groups 2&3) and ChABC (green; group 4) animals. Additional and normalizedata shown in Fig. 5. Data presented in (*B*) are normalized (ipsilateral/contralateral results from the same animal) to control for variance of all extenuating factors. Data assessed via ANOVA with normalized sample size of groups = 5. Sample sizes raw data: control ipsi = 5; control contra = 5; injury ipsi = 6; injury contra = 6; ChABC ipsi = 5; ChABC contra = 5. ^*^ *P* < 0.05, ^**^ *P* < 0.01 and ^***^ *P* < 0.001. If no *post hoc* result is shown, the comparison was not significant. Values represent the mean ± SD. Triangle data points represent group 3 animals. Abbreviations: Ipsi, ipsilateral; contra, contralateral hemidiaphragm recordings. [Color figure can be viewed at wileyonlinelibrary.com]

We demonstrated that global and local alterations to the ipsilateral hemidiaphragm following injury‐induced paralysis facilitate a continuous supply of oxygen to the muscle, with a modest trend for increased supply to the contralateral hemidiaphragm (Fig. 5 *B–H*; see also Appendix, Fig. A5*A* and *B*). This continuous supply of fuel to the ipsilateral muscle facilitates its capacity to yield power despite prolonged paralysis. However, despite morphological alterations to the muscle, the oxygen demand significantly decreases following paralysis (ANOVA, Type I: *P* = 0.018, *F*_2,12_ = 5.77; Type IIa: *P* = 0.018, *F*_2,12_ = 5.77; Type IIb/x: *P* = 0.018, *F*_2,12_ = 5.77; whole diaphragm: *P* = 0.050, *F*_2,12_ = 3.87) (Fig. 5 *I*; see also Appendix, Fig. A5*C*). Critically, following restoration of diaphragm function, oxygen supply to both the ipsilateral and contralateral hemidiaphragms increased (ANOVA, Type I: *P* < 0.0001, *F*_5,26_ = 16.4; Type IIa: *P* < 0.0001, *F*_5,26_ = 17.4; Type IIb/x: *P* < 0.0001, *F*_5,26_ = 12.6) (Fig. 5 *B–H*; see also Appendix, Fig. A5*A* and *B*). This confirms that local alterations to capillary supply through structural remodelling facilitated an increase in oxygen supply capacity to the muscle across all fibre types as a result of the increased work being performed in recovery. Importantly, however, the tissue demand for oxygen does not recover following ChABC‐mediated restoration of diaphragm function (Fig. 5 *C–I*; see also Appendix, Fig. A5*C*).

## Discussion

Having demonstrated that robust respiratory motor recovery is possible at chronic timepoints following severe SCI, we now provide a comprehensive assessment of the mechanisms facilitating rapid diaphragm recovery. We show that lateralized cervical SCI induces paralysis in the ipsilateral hemidiaphragm. Importantly, this effect causes muscle hypertrophy. This, combined with alterations to the muscle's global and local morphology, facilitates a maintained hemidiaphragm microvascular network, but reduced capacity to utilize oxygen within the tissue. These morphological changes foster a metabolic strain within the system, and mediate alterations in muscle function, shifting the optimal properties beyond that typical for breathing. However, we show that the retained microvascular supply facilitates hemidiaphragm capacity to function despite paralysis, which can be rapidly harnessed following neural reconnections at the spinal level. Importantly, this recovery occurs rapidly in all treated animals following chronic injury using a single injection of ChABC within the PMP. Indeed, this treatment works better at chronic time points rather than when applied acutely after injury. We determine that return of activity in the previously paralysed hemidiaphragm mediates a reduction in size of muscle fibres and alterations to the local tissue morphology that recover the capacity to deliver oxygen to the tissue. Critically, we show that this also normalized optimal functional properties of the muscle. However, this recovery is incomplete. The diaphragms oxidative demand is still reduced following recovery, this prevents complete restoration of normal functionality as shown by the hemidiaphragm EMG recordings. Our holistic approach shows that, despite the presence of diaphragm activity following spinal recovery, the system is not yet operating optimally. We theorize that the metabolic deficit of respiratory muscles must be recovered to achieve complete motor restoration.

We used a unique combination of advanced techniques to determine the holistic effects of spinal‐induced paralysis and recovery on diaphragm activity, functional capacity, phenotype, morphology, microvascular supply and oxygen transport. This comprehensive, physiologically relevant approach has never been used to assess the activity of the diaphragm. We have shown that the subtle changes detected through the more commonly used isometric/isotonic methods, manifest into substantial alterations in muscle functional mechanics, when assessed using the more physiologically appropriate work loop technique (Altringham & Young, 1991). Similarly, through a comprehensive analysis of local muscle morphology and associated biophysical properties, a more complete assessment of paralysis and recovery upon the diaphragm can be determined. Our work highlights the need to utilize more realistic methods for determining muscle functional properties, aiming to effectively assess changes following trauma and (in this instance) successful recovery.

Critically, we perform this in a SCI model that produces a permanent paralysis of the hemidiaphragm, allowing the full effects of injury to be assessed. Some previous studies have demonstrated spontaneous recovery of ipsilateral hemidiaphragm function in ∼50% of animals in as little as 2 weeks following C2 hemisection (Mantilla *et al*. 2012). We assume that alterations in methodology account for these discrepancies as it has not been demonstrated within our model of this injury (Warren *et al*. 2018*b*). Similarly, these differences in injury effect may account for the lack of change demonstrated within the ventilatory parameters of animals shown within our data. Such a result may be expected because these parameters (assessed via EMG) reflect total ventilatory performance, and not the paralysis of one respiratory muscle as occurs in this injury model (Warren *et al*. 2018*b*) or demonstrated following nerve denervation (Khurram *et al*. 2017). Astonishingly, despite chronic paralysis, the diaphragm retained capacity to produce power, although it would not function optimally for breathing. The diaphragm typically demonstrates signs of atrophy within 24 h after spinal hemisection (Gill *et al*. 2014), although the degree of denervation and atrophy is known to vary depending on the type of injury and muscle involved (Biering‐Sorensen *et al*. 2009). It is possible that the full effects of SCI have not been realized within the hemidiaphragm over the time‐course described in the present study. However, as we demonstrate significant alterations in diaphragm functional capacity, global morphology, capillary domain size and oxygen dynamics, this would suggest that paralysis has had a profound effect on the muscle. Indeed, we have shown that diaphragm paralysis from this injury can last up to 1.5 years following trauma but still recover function within days of ChABC application (Warren *et al*. 2018*b*), indicating that functional capacity of the hemidiaphragm is retained regardless of the length of time muscle paralysis has occurred. Nonetheless, we demonstrate here that, although the muscle may function following neurological restoration, it may not do so optimally, reducing functional capacity and recovery. Gender effects on respiratory function following SCI, diaphragm kinetics and the muscles morphological properties are modest (Alilain & Goshgarian, 2008; Mantilla *et al*. 2012; Greising *et al*. 2015; Khurram *et al*. 2018). As such, it is probable that our data are applicable across genders.

A previous study assessed diaphragm muscle kinetics 6 weeks following C2 hemisection within a group of animals where ∼40% showed spontaneous activity within the muscle (Mantilla *et al*. 2013*b*). They demonstrated a reduction in specific force following injury and a modest reduction in cross‐sectional area of diaphragm Type IIx and/or IIb fibres. These data are distinct from those that we describe. However, this is not unexpected considering profound methodological differences between these studies and functionally different outcomes between the groups assessed.

Recovery of respiratory motor function at chronic stages following experimental spinal injury has typically been limited either by the proportion of animals responding to treatments or only modest beneficial effects being induced (Nantwi & Goshgarian, 1998; Golder & Mitchell, 2005; Warren *et al*. 2018*b*). Here, we show that ChABC mediated respiratory mediated motor recovery can occur only at chronic time points following injury. The pathway for this re‐connection has not been addressed within the present study. Recovery only chronically following injury may be a result of the remodelling of existing circuitry (Dietz, 2010; Buttry & Goshgarian, 2014) or sprouting/recruitment of new serotonergic, V2a or glutamatergic interneuronal pathways from the contralateral respiratory groups (Porter, 1895; Lane *et al*. 2008*b*; Cregg *et al*. 2017; Zholudeva *et al*. 2018; Warren *et al*. 2018*b*). Furthermore, latent pathways (Porter, 1895) may only be activated at chronic time points following trauma. Nonetheless, in our model, the removal of CSPG from the surrounding perineuronal nets and extracellular matrix is critical to enabling any functional recovery of the motor system. Without this removal, recovery does not occur (Warren & Alilian, 2018; Warren *et al*. 2018*b*). The mechanistic cause of plasticity‐induced recovery only at chronic time points in this model is something that we are actively pursuing. This includes the assessment of diaphragm muscle kinetics and local morphology at acute time points following injury to assess the degree that the peripheral muscle plays in systemic function.

There is a substantial decline in hemidiaphragm function at chronic stages after SCI induced paralysis during normal breathing, caused by significant alterations in the optimization of the muscle kinetics. This was mediated through alterations in muscle morphology, including hypertrophy of slow muscle fibres that (at the same time as facilitating muscle power generation) decreased vascularization of the tissue. However, the functional capacity of the diaphragm was not lost. Once treatment re‐established neurological input to the respiratory motor pools, diaphragm activity was restored to optimal working conditions. Importantly, this explains how the diaphragm can recover normal working mechanics rapidly and robustly following reconnection of spinal pathways (Warren *et al*. 2018*b*), as well as retain fatigue resistance, because the peripheral muscle was able to rapidly revert to normal functional characteristics. This was mediated largely through a restoration of muscle global and local morphological characteristics. These effects demonstrate that the diaphragm will not only remodel in responses to paralysis, injury or disease, but also has the capacity to revert to control conditions facilitating respiratory motor recovery. Our assessment of oxygen transport modelling showed that the structural changes in muscle morphology and vascular supply following injury and recovery maintained/improved the capacity to deliver oxygen and fuel to the muscle, although not the capacity to utilize it, which would continue to negatively impact functionality. It is potentially for this reason that the functional properties and EMG activity of the diaphragm are not fully restored to control values following ChABC treatment. We hypothesize that these deficits within the tissue would need to be restored to achieve complete functional motor recovery following SCI.

The citrate synthase assay was used as an index of differential oxygen consumption within the diaphragm. Most readily available measures of this activity have limitations as all assess maximal mitochondrial function rather than values processed typically within a tissue. However, the citrate synthase assay was used in the development of the oxygen modelling software (Al‐Shammari *et al*. 2019), following the same preparation and methodology used in the present study and was developed to accommodate these factors. As such, the citrate synthase assay was considered the most appropriate assessment to reliably index hemidiaphragm differential oxygen consumption. One could infer from the citrate synthase data that spinal ChABC treatment caused a substantive decline of mitochondrial activity in the diaphragm. However, the ChABC treated animals in group 3 did not yield similar data to group 4 (Fig. 5 *A*). As such, the decrease in citrate synthase activity was probably caused by the recovery of function in the muscle rather than being treatment specific. Furthermore, our previous data have shown that animals with ChABC‐mediated respiratory motor recovery have an increase in metabolic flux, which would compensate for any reduction in oxygen consumption (Warren *et al*. 2018*b*).

It is possible that other skeletal muscles would respond in a similar way to prolonged injury induced paralysis and recovery of activity as we have shown in the diaphragm. Evidence suggests that skeletal muscles respond with similar morphological and microvascular alterations over a comparable time frame following challenge (Corpeno *et al*. 2014). However, these muscles respond differently to ageing, exercise and disease with respect to their functional properties, morphology and capillarity (Tallis *et al*. 2014; Bowen *et al*. 2017). The present study, using a robust model of cervical SCI to impair diaphragm function, describes the importance of microvascular supply and metabolic status on the functional capacity and motor activity of skeletal muscle. We have previously described a shift in the phenotype of type II fibres from oxidative to glycolytic and a loss in capillaries within the tibialis anterior muscle using a chronic, thoracic spinal contusion model (Kissane *et al*. 2018*a*). Training and epidural stimulation of the paralysed muscle facilitated normalization of tissue morphology and correlated with improved locomotor function (Kissane *et al*. 2018*b*), although assessment of functional characteristics and biophysical properties was not as comprehensive as described here. These data demonstrate that muscles within different motor systems present alterations in global and local morphology following paralysis that can be restored after motor recovery. Furthermore, it suggests that muscle movement *per se* (i.e. muscle lengthening/shortening) can help maintain characteristics within the paralysed/recovering muscle. We hypothesize that constant passive movement of the paralysed hemidiaphragm allows maintenance of muscle oxygen transport, delaying any effects of atrophy, and provides the capacity to retain function described herein.

Passive movement poses great therapeutic potential in clinical populations as shown in peripheral vascular disease (Høier *et al*. 2013), mechanically ventilated ICU patients (Llano‐Diez *et al*. 2012) and chronic SCI sufferers (Rayegani *et al*. 2011). Passive movement of skeletal muscles has been shown to increase blood flow and, subsequently, oxygen supply within the tissue (Hellsten *et al*. 2008). Furthermore, increases in interstitial vascular endothelial growth factor, endothelial nitric oxide synthase mRNA and matrix‐metalloprotease expression produced by the movement are integral to the preservation or augmentation of the microvasculature (Egginton *et al*. 2011). We have shown these effects within the diaphragm following injury induced paralysis, suggesting that passive stretch may be the cause. Furthermore, passive stretch has been shown to have negligible effect upon preservation of the metabolic processes or haemodynamics within muscle (Hellsten *et al*. 2008), effects that are echoed with our data. Continuous passive movement of the paralysed ipsilateral hemidiaphragm (caused by contraction of the contralateral muscle) (Warren *et al*. 2018*b*) would occur at the same frequency as eucapnic breathing, maintaining the heterogeneous fibre type composition (Egginton *et al*. 2011) and preserving (if not slightly increasing) the microvascular supply in the diaphragm (Høier *et al*. 2010). This acts to maintain the hemidiaphragm endothelial cell integrity following injury and recovery and thus shear stress through continuous perfusion of the tissue microcirculation. These are all effects demonstrated within our data. Passive movement of the ipsilateral hemidiaphragm also probably aids maintenance of muscle function through the addition of sarcomeres (Wang & Ramirez‐Mitchell, 1983; Lynn & Morgan, 1994). This would lead to a rightward shift in the force–length relationship, whereas uneven addition of sarcomeres throughout the muscle would cause a broadening of the force–length relationship plateau (Huijing *et al*. 1994). This would explain the increased power generated at higher cycle frequencies shown by the ipsilateral hemidiaphragm following injury induced paralysis and ChABC‐mediated recovery in our data. This suggests that passive motion is a fundamental mechanism for the maintenance of diaphragm global morphology and functional characteristics and thus key to the rapid restoration of respiratory motor activity following injury. Activities such as passive movement and prolonged standing have shown to facilitate substantial recovery in humans following SCI (Rayegani *et al*. 2011), suggesting that this mechanism of maintenance could act over a variety of skeletal muscles to aid functional restoration. Our data support the use of passive movement as a therapeutic tool (Huijing *et al*. 1994; Jeong *et al*. 2011; Fowler *et al*. 2019), which may be exploited to facilitate complete recovery of motor function following all forms of SCI.

Our data have shown that phenotypical and morphological changes to the diaphragm appear to occur simultaneously (Miyata *et al*. 1995). However, metabolic alterations in the muscle do not alter concurrently with alterations to capillary domain size following SCI‐induced paralysis and recovery of activity. The persistent reduction in metabolic activity within the diaphragm may cause dysfunction in the system, meaning that complete restoration of motor function cannot occur. Indeed, a centrally applied therapy would not be expected to specifically alter metabolic function in the periphery because unchallenged breathing alone will probably not restore mitochondrial activity. Modelling physiologically appropriate oxygen consumption using histology‐derived structural morphometrics has provided the most comprehensive overview of diaphragm O_2_ dynamics in the context of normal, SCI and recovered states. This is important because, in normal, non‐pathological tissue, there is a maintenance of the relationship between metabolic capacity and microvascular supply (Barnouin *et al*. 2017). However, dysregulation occurs when the system is under stress or diseased; for example, with pulmonary arterial hypertension (Fowler *et al*. 2019). The sustained capacity to deliver oxygen and remove waste products within the diaphragm following SCI is offset by the reduced capacity to utilize oxygen for fatty acid oxidation. There is no indication that this would improve with time following ChABC‐mediated recovery of activity, despite increases in muscle EMG activity. As such, we propose this as a novel target to recover complete diaphragm function following paralysis by applying treatment to the muscle that would restore mitochondrial function. It is possible that treatments for SCI altering systemic metabolic activity (e.g. calorific restriction or reductions in glycolytic function) (Jeong *et al*. 2011; Graham *et al*. 2019) have been successful in part because of the effect that they have had on peripheral muscle. This indicates that combining treatments that (i) aid functional plasticity and connectivity at the spinal level; (ii) maintain capillarity though passive stretch; and (iii) increase oxidative phosphorylation in paralysed muscles (e.g. idebenone or dimethylglycine) (Liet *et al*. 2003) may provide an effective treatment strategy for complete recovery from such trauma.

We have employed a comprehensive set of advanced techniques to provide a holistic overview of the changes to the diaphragm following SCI induced paralysis and ChABC‐mediated restoration of activity. Our data show the speed with which activity to a chronically paralysed muscle can be restored in all animals by evoking central plasticity. Following paralysis, the diaphragm shows distinct global and local morphological alterations, modifying its functional characteristics from optimal levels. However, through successful maintenance of tissue oxygen supply, the diaphragm retains the capacity, upon restoration of activity, to function at near normal capacity. Although a dysregulation remains with respect to the metabolic status of the muscle, we propose this as a novel target for maximizing complete motor recovery following reconnection of spinal pathways. This is not only of consequence to the treatment of SCI patients, particularly living at chronic stages following trauma, but also relates to other muscle wasting disorders, suggesting the potential universal applicability of conclusions in multiple motor systems and conditions. Our data demonstrate that the effects of any trauma are systemic, and thus the importance of treating SCI both centrally and peripherally to achieve maximal functional effects could be common to the treatment of all affected motor systems.

## Additional information

### Competing interests

The authors declare that they have no competing interests.

### Author contributions

PMW and RWPK were responsible for the animal work, data acquisition, tissue processing, immunohistochemistry, imaging and data analysis. PMW was responsibe for article preparation. All of the authors contributed to the article editing. PMW and RWPK conceived and designed the project under the advice of JCFK, SE and GNA. All authors have approved the final version of the article submitted for publication, agree to be accountable for the work and qualify for authorship.

### Funding

This work was financially supported by Wings for Life (WFL‐UK‐008/15); the European Union (Operational Programme Research, Development and Education) in the framework of the project ‘Centre of Reconstructive Neuroscience’ (CZ.02.1.01/0.0./0.0/15_003/0000419) to JCFK; MRC project grant (MR/S011110/1) to JCFK and PMW; and the King's College London Prize Fellowship to PMW.

## Supporting information


**Statistical Summary Document**

## Data Availability

Data are available from the corresponding author upon reasonable request.

## Appendix

**Table A1:** Isometric characteristics of the contralateral and ipsilateral hemidiaphragms in control (group 1), injured (groups 2&3) and ChABC (group 4) treated animals

	Control		Injury		ChABC treated		*P* value
	*Contra*	*Ipsi*	*Contra*	*Ipsi*	*Contra*	*Ipsi*	
Optimal muscle length, *L*_0_ (m)	0.021 ± 0.002	0.020 ± 0.002	0.024 ± 0.002	0.021 ± 0.002	0.026 ± 0.002	0.020 ± 0.002	< 0.0001
Twitch‐to‐tetanus ratio	0.33 ± 0.056	0.34 ± 0.068	0.37 ± 0.060	0.39 ± 0.054	0.38 ± 0.049	0.44 ± 0.034	0.024
Maximal isometric tetanic stress (kN m^−2^)	240.8 ± 32.5	238.4 ± 41.7	230.9 ± 35.4	237.1 ± 45.4	215.6 ± 45.1	265.0 ± 27.3	0.508
Twitch rise time (s)	0.021 ± 0.002	0.021 ± 0.003	0.020 ± 0.001	0.019 ± 0.002	0.021 ± 0.002	0.021 ± 0.003	0.782
Twitch half relaxation time (s)	0.030 ± 0.005	0.031 ± 0.007	0.028 ± 0.003	0.026 ± 0.002	0.032 ± 0.009	0.028 ± 0.005	0.363
Sample size	10	12	6	7	5	5	

Values shown are the mean ± SD. Data assessed via ANOVA.

**Table A2:** Isotonic properties of the contralateral and ipsilateral hemidiaphragms in control (group 1), injured (groups 2&3) and ChABC (group 4) treated animals

	Control		Injury		ChABC treated		*P* value
	*Contra*	*Ipsi*	*Contra*	*Ipsi*	*Contra*	*Ipsi*	
*V*_max_ (*L*_0_s^−1^)	8.87 ± 1.05	9.14 ± 1.19	8.70 ± 0.311	10.13 ± 1.45	11.21 ± 2.15	10.28 ± 0.75	0.008
Power ratio	0.097 ± 0.01	0.092 ± 0.03	0.098 ± 0.01	0.079 ± 0.03	0.085 ± 0.01	0.097 ± 0.01	0.497
Force at max power (*P*/*P*_0_)	0.32 ± 0.03	0.33 ± 0.03	0.30 ± 0.02	0.32 ± 0.02	0.32 ± 0.02	0.35 ± 0.02	0.063
Velocity at max power (*L*_0_s^−1^)	2.72 ± 0.35	2.75 ± 0.40	2.82 ± 0.43	2.83 ± 0.42	2.91 ± 0.25	2.88 ± 0.42	0.939
Max isotonic power (${\dot{W}}_{\max}$; W kg^−1^)	196.2 ± 43.4	202.4 ± 46.4	186.9 ± 34.6	210.4 ± 56.5	190.5 ± 39.9	252.1 ± 47.1	0.226
*V*/*V*_max_ at maximal power	0.31 ± 0.02	0.30 ± 0.01	0.32 ± 0.04	0.28 ± 0.03	0.26 ± 0.04	0.28 ± 0.04	0.073
Sample size	10	11	6	7	5	5	

Values shown mean ± SD. Data assessed via ANOVA. Definitions: *V*_max_, the maximal shortening velocity; *V/V*_max_, ratio defining the velocity that generates maximal isotonic power compared to *V*_max_; *P/P*_0_, ratio defining the force that generated maximal isotonic power divided by maximal isometric force; ${\dot{W}}_{\max}$, the peak instantaneous isotonic power; and power ratio, [*W*_max_/(*P*_0_.*V*_max_)].
